# SMRT and Illumina RNA sequencing reveal novel insights into the heat stress response and crosstalk with leaf senescence in tall fescue

**DOI:** 10.1186/s12870-020-02572-4

**Published:** 2020-08-03

**Authors:** Yiguang Qian, Liwen Cao, Qiang Zhang, Maurice Amee, Ke Chen, Liang Chen

**Affiliations:** 1 Shenzhen Key Laboratory of Environmental Chemistry and Ecological Remediation, College of Chemistry and Environmental Engineering, Shenzhen University, Shenzhen, People’s Republic of China; 2 CAS Key Laboratory of Plant Germplasm Enhancement and Specialty Agriculture, Wuhan Botanical Garden, The Innovative Academy of Seed Design, Chinese Academy of Sciences, Wuhan, People’s Republic of China; 3 College of Resources and Environmental Science, Key Laboratory of Catalysis and Energy Materials Chemistry of Ministry of Education & Hubei Key Laboratory of Catalysis and Materials Science, South-Central University for Nationalities, Wuhan, People’s Republic of China; 4 Center of Economic Botany, Core Botanical Gardens, Chinese Academy of Sciences, Wuhan, People’s Republic of China

**Keywords:** Tall fescue, Short- and long-term heat stress, Senescence, SMRT and Illumina RNA-Seq, Responsive genes, Alternative splicing

## Abstract

**Background:**

As a cool-season grass species, tall fescue (*Festuca arundinacea*) is challenged by increasing temperatures. Heat acclimation or activation of leaf senescence, are two main strategies when tall fescue is exposed to heat stress (HS). However, lacking a genome sequence, the complexity of hexaploidy nature, and the short read of second-generation sequencing hinder a comprehensive understanding of the mechanism. This study aims to characterize the molecular mechanism of heat adaptation and heat-induced senescence at transcriptional and post-transcriptional levels.

**Results:**

Transcriptome of heat-treated (1 h and 72 h) and senescent leaves of tall fescue were generated by combining single-molecular real-time and Illumina sequencing. In total, 4076; 6917, and 11,918 differentially expressed genes (DEGs) were induced by short- and long-term heat stress (HS), and senescence, respectively. Venn and bioinformatics analyses of DEGs showed that short-term HS strongly activated *heat shock proteins* (*Hsps*) and *heat shock factors* (*Hsfs*), as well as specifically activated *FK506-binding proteins* (*FKBPs*), calcium signaling genes, glutathione S-transferase genes, photosynthesis-related genes, and phytohormone signaling genes. By contrast, long-term HS shared most of DEGs with senescence, including the up-regulated chlorophyll catabolic genes, phytohormone synthesis/degradation genes, stress-related genes, and *NACs*, and the down-regulated photosynthesis-related genes, *FKBPs*, and *catalases*. Subsequently, transient overexpression in tobacco showed that *FaHsfA2a* (up-regulated specifically by short-term HS) reduced cell membrane damages caused by HS, but *FaNAC029* and *FaNAM-B1* (up-regulated by long-term HS and senescence) increased the damages. Besides, alternative splicing was widely observed in HS and senescence responsive genes, including *Hsps*, *Hsfs*, and phytohormone signaling/synthesis genes.

**Conclusions:**

The short-term HS can stimulate gene responses and improve thermotolerance, but long-term HS is a damage and may accelerate leaf senescence. These results contribute to our understanding of the molecular mechanism underlying heat adaptation and heat-induced senescence.

## Background

Tall fescue (*Festuca arundinacea*) is one of the most important and widely used cool-season turfgrass and forage species all over the world [1]. The optimum temperature for tall fescue growth ranges from 15 to 25 °C. The high temperature will affect its growth and development [2, 3]. One of the major symptoms of heat damage is premature leaf senescence. Leaf senescence is a critical concern for turfgrass because it negatively influences not only the forage production, but also aesthetic turf quality. However, because of increasing global temperature, an extreme high temperature greater than 35 °C during summer is more frequent and extensively distributed. Heat stress (HS) has been a major threat for tall fescue.

As sessile organisms, plants are compelled to develop diverse systems to prevent damages caused by high temperatures [4]. The induction of heat shock proteins (Hsps), including Hsp100s, Hsp90s, Hsp70s, Hsp60s, and small Hsps under the control of heat stress transcription factors (Hsfs) has been reported to play a pivotal role in the heat stress response (HSR) and in acquired thermotolerance in plants [5–7]. In *Arabidopsis thaliana*, the complex transcriptional networks of HS in HsfA1-dependent or independent pathways, have been elucidated [8]. Reactive oxygen species (ROS) scavenging enzymes and plant hormones were also shown to participate in heat stress response [9–11]. However, investigation on regulatory networks during HSR was mainly focused on model plants *Arabidopsis* and tomato. For tall fescue, most studies were about genome-wide or certain gene family expression profiles of HSR by transcriptional analysis. The expression levels of *FaHsfs*, especially *FaHsfA2s*, and the target genes of *FaHsfs*, including *Hsps*, *ascorbate peroxidase* (*APX*), *inositol-3-phosphate synthase* (*IPS*), and *galactinol synthase* (*GOLS1*) were significantly up-regulated by HS [12]. In addition, genes involved in cell division, cell cycle, cell maintenance, photosynthesis, protein synthesis, signaling, metabolism, hormone metabolism, and DNA, RNA and protein degradation were dramatically responsive to HS in tall fescue with opposite thermotolerance or with different heat treatments [13–15]. However, because of the complex genome background and the difficulty in developing the transgenic system, functional analysis of *Hsfs*, *Hsps* or heat-related genes in tall fescue has been hardly reported [16, 17]. Only one *FaHsfA2c* was demonstrated to enhance heat tolerance of transgenic tall fescue through activating *Hsps* [16]. Although the research on HSR in tall fescue has advanced persistently in recent years, the similarity and difference between short- and long-term HSRs in tall fescue are still unclear.

As the major symptom of heat injury, heat-induced leaf senescence in cool-season grass species is of wide concern. Heat-induced leaf senescence was positively associated with ethylene and abscisic acid (ABA), and negatively associated with cytokinin synthesis in Bentgrass, and it can be suppressed through a transformation with cytokinin synthesis gene *IPT* [18, 19]. The metabolic and protein profiling identified that the content of certain acids, sucrose, and monosaccharides, proteins in photosynthesis, and amino acid metabolism were related to the alleviation of heat-induced senescence by cytokinin and ethylene inhibitor [20, 21]. However, all these studies were performed in Bentgrass, and the relationship between heat stress response and leaf senescence at the transcriptional level in tall fescue is still unclear.

In addition to transcriptional changes, post-transcriptional changes generated by alternative splicing (AS) also play important roles in HSR and senescence [22–25]. HS-induced AS has been demonstrated as a common feature among *Hsfs* in *Arabidopsis* [26]. HS post-transcriptionally regulated the expression of *HsfA2* by AS. However, there is still no study about the role of AS in heat stress response in tall fescue. Furthermore, the role of AS in senescence response has only been studied in animals and humans but not in plants. Therefore, the genome-wide response of AS to HS and HS-induced senescence in tall fescue is worth exploring.

Tall fescue is an allohexaploid (2n = 42, PPG1G1G2G2) with a genome size of about 5.50 million Kb. The P genome comes from *F. pratensis* and the G1G2 genome is from *F. arundinacea* var. glaucescens auct [27]. The highly complex genome constitution, the absence of reference genome sequences, and the short-read length of Illumina sequencing have posed large challenges for exploring HS and senescence response in tall fescue by second-generation sequencing technology. Single-molecular real-time (SMRT) sequencing which generates full-length transcripts and constructs reference unigenes directly without further assembly can resolve these difficulties [28]. Also, SMRT sequencing technology can identify alternative isoforms produced by AS with higher accuracy [29]. In this study, we aimed to investigate the similarity and difference between short- and long-term HSR, and explore the relationship between HS and leaf senescence in tall fescue at the transcriptional and post-transcriptional level. Therefore, both Pacific Biosciences (PacBio) SMRT and Illumina-based sequencing of the transcriptome of ‘Houndog 5’ under control (Con), 1 h of heat treatment (HT_1h), 72 h of heat treatment (HT_72h), and natural senescence (Sen) conditions were performed. Our results help elucidate the underlying mechanism of HSR and heat-accelerated leaf senescence in tall fescue, and can provide important clues for in-depth characterization of heat-resistance breeding candidate genes in tall fescue.

## Results

### Effects of heat stress and senescence on physiological indexes

**Fig. 1:**
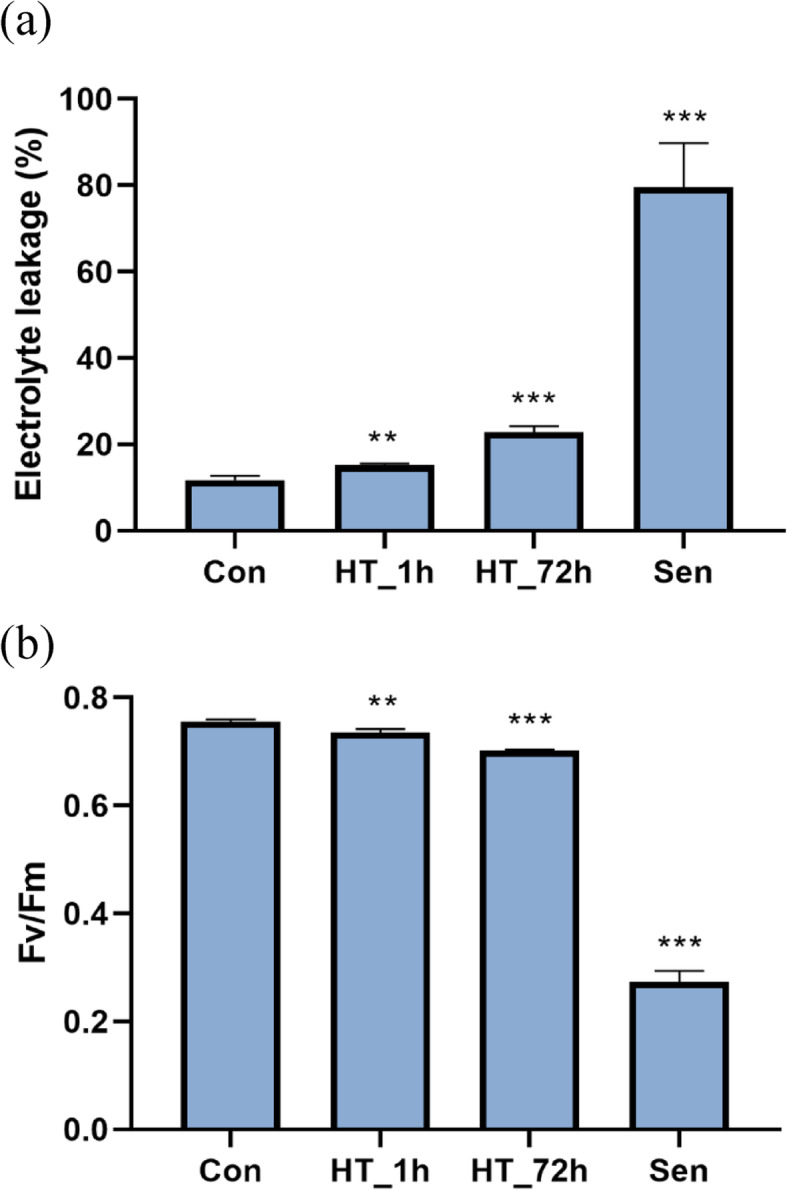
Physiological variations induced by heat stress and senescence in tall fescue. **a**, **b** Electrolyte leakage (EL) (a) and PSII max-photo efficiencies (Fv/Fm) (**b**) of Con, HT_1h, HT_72h and Sen. Asterisks indicated significant difference with wild type at high temperature as estimated using Student’s t-test (** *P* < 0.01, *** *P* < 0.001). Error bars indicate the SD of three biological repeats. Con, control; HT_1h, 1 h of 38 °C heat treatment; HT_72h, 72 h of 38 °C heat treatment; Sen, senescent leaves

To determine the physiological changes caused by heat stress and senescence, electrolyte leakage (EL), and Fv/Fm were measured under control, short-term HS, long-term HS, and natural senescence conditions, respectively (Fig. 1). The EL is used to represent cell membrane damage. As expected, EL was increased with the increment of heat treatment time. The EL of senescent leaves was significantly higher than that in control green leaves as well. Fv/Fm reflects the maximum quantum efficiency of photosystem II (PSII) photochemistry. After long-term heat treatment, Fv/Fm was significantly decreased. Likewise, Fv/Fm significantly declined in senescent leaves.

### A global description of full-length transcriptome

**Fig. 2:**
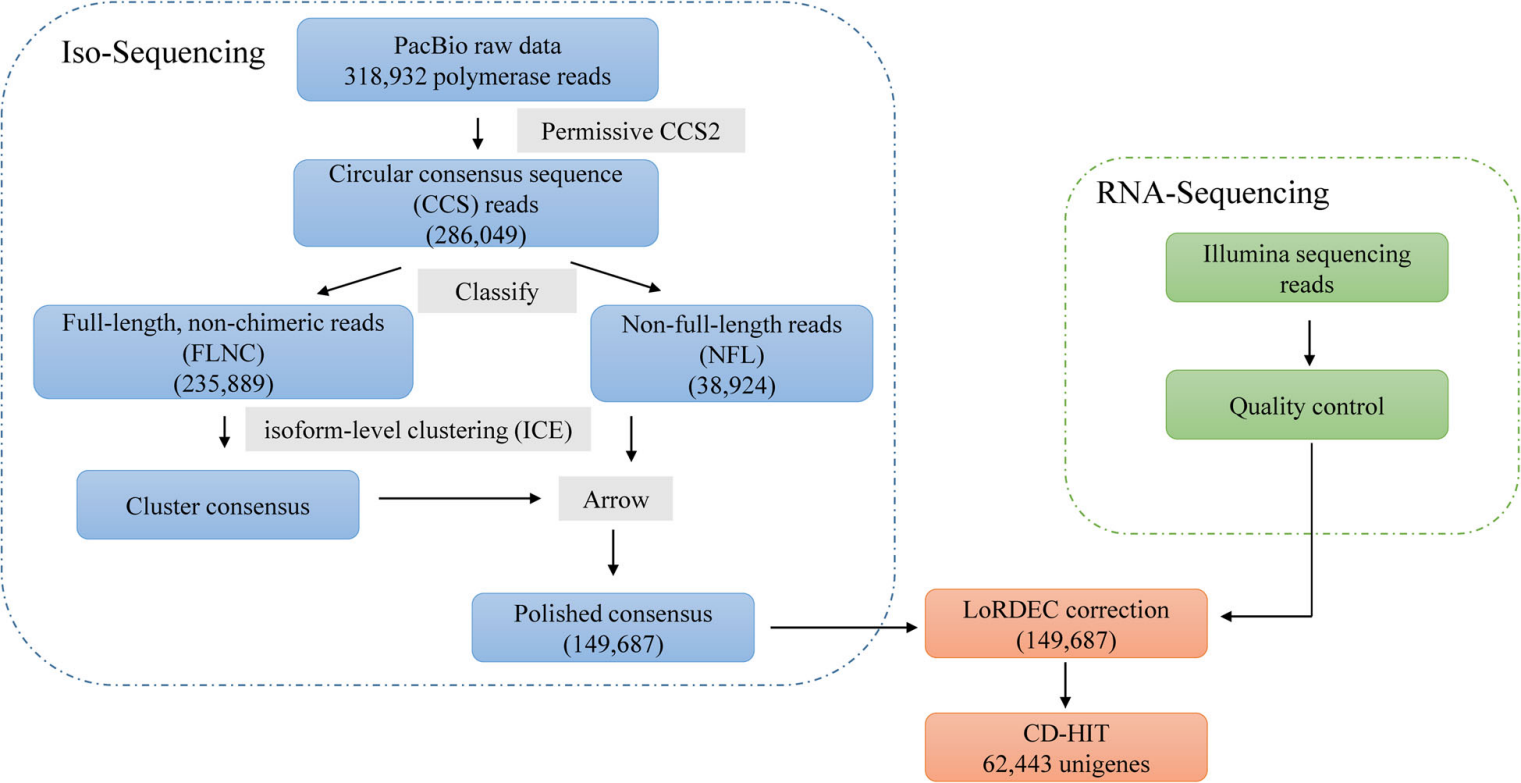
The process and characteristics of full-length cDNA sequencing in tall fescue. CCS, circular consensus sequence; FLNC, full-length, non-chimeric; NFL, non-full-length

To comprehensively investigate variation in expression of genes responsive to HS and senescence in tall fescue, we sequenced 12 mRNA samples from leaves of ‘Houndog 5’ at different developmental stages or under different times of HS treatments using Illumina HiSeq platform (second-generation sequencing). To obtain a more comprehensive reference transcriptome in absence of a reference genome of tall fescue, full-length mRNA sequencing of mixed tall fescue RNA derived from above 12 mRNA samples was conducted on the PacBio RSII platform (Fig. 2). A total of 318,932 polymerase reads were generated (PRJNA647166). After filtering adaptors and low-quality reads (less than 50 bp), 11,961,314 subreads with a mean length of 1355 bp were obtained. Then, all the subreads were processed into 286,049 circular consensus sequences (CCS) reads to further improve the data quality. Depending on whether 5′ primer, 3′ primer, and poly (A) sequences were detected, 235,889 full-length non-chimeric reads (FLNCs) were extracted from the CCS reads. These FLNCs were subsequently corrected using Iterative Clustering for Error Correction (ICE), LoRDEC and CD-HIT to eliminate up to 99.99% of sequencing errors. Finally, 62,443 unigenes with an average length of 2595 bp, N90 of 1282 bp were successfully identified, and 23,310 of the unigenes were longer than 3000 bp.

To analyze the function of the 62,443 unigenes of tall fescue, functional annotations of all full-length isoforms were investigated using seven databases, including non-redundant protein sequence database (Nr), nucleotide sequence database (Nt), Pfam, clusters of orthologous groups of proteins (KOG/COG), Swiss-prot, Kyoto encyclopedia of genes and genomes (KEGG), and gene ontology (GO). In total, there were 55,075 unigenes (88.20%) successfully annotated in at least one database, with 19,677 unigenes (31.51%) in all seven databases (Additional file 1).

For GO analysis (Additional file 2), genes involved in ‘metabolic process’ (15,300), ‘cellular process’ (14,564), and ‘single-organism process’ (8587) were highly represented in biological process. In terms of cellular component category, ‘cell’ (5842), ‘cell part’ (5842), and ‘organelle’ (3875) were highly enriched. Within the molecular function category, the major sub-categories were ‘binding’ (19,433), ‘catalytic activity’ (14,076), and ‘transporter activity’ (1583).

For KOG analysis (Additional file 2), a total of 32,982 enriched unigenes were divided into 26 groups. The largest group was ‘general function prediction only’ (7157), followed by ‘posttranslational modification, protein turnover, chaperones’ (6310), and ‘signal transduction mechanisms’ (3536).

For KEGG analysis (Additional file 2), 50,614 unigenes were classified into six main biochemical pathways: ‘cellular processes’, ‘environmental information processing’, ‘genetic information processing’, ‘metabolism’, ‘organismal systems’, and ‘human diseases’. Within the metabolism pathway, ‘energy metabolism’, ‘carbohydrate metabolism’, and ‘global and overview maps’ were prominently represented. The pathways related to ‘organismal systems’ included ‘endocrine system’, ‘immune system’, ‘aging’, and ‘environmental adaptation’. All functional annotations provided important information for analyzing the processes and pathways involved in HSR and senescence.

### Alternative splicing analysis in heat stress response and senescence

**Fig. 3:**
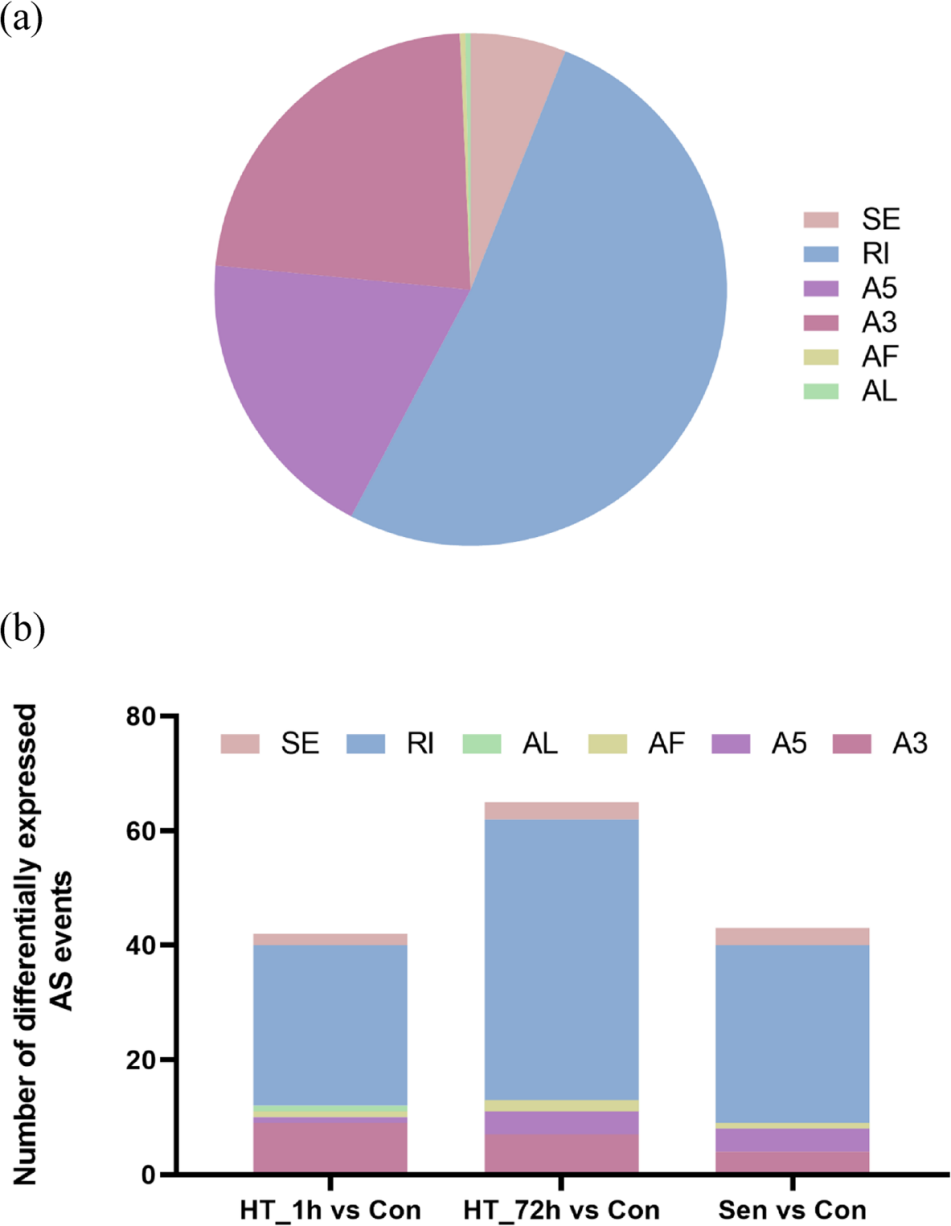
Alternative splicing (AS) events analysis after heat stress treatments and natural senescence. **a** The number of six AS events in isoform sequencing (Iso-seq) sample. **b** The number of differentially expressed AS events in HT_1h vs Con, HT_72h vs Con, and Sen vs Con. SE, skipping exon; MX, mutually exclusive exon; A5, alternative 5′ splice-site; A3, alternative 3′ splice-site; RI, retained intron; AF, alternative first exon; AL, alternative last exon

One of the most important features of PacBio sequencing is the ability to identify AS. Given that there was no reference genome information of tall fescue, we utilized the COGENT to partition non-redundant transcripts into putative gene families and reconstruct each family into one or several full-length UniTransModels for further AS analysis. Of the 62,443 transcripts, COGENT constructed 6297 gene families with two or more isoforms. After splicing junction analysis, 3537 genes were identified with AS, generating 8312 isoforms. Then, a total of 298 AS events, including skipping exon (SE), mutually exclusive exon (MX), alternative 5′ splice-site (A5), alternative 3′ splice-site (A3), retained intron (RI), alternative first exon (AF), and alternative last exon (AL) were detected with SUPPA. RI was the most abundant AS events in tall fescue followed by A3 (Fig. 3a). Subsequently, the differential expression of AS events was analyzed. A total of 42, 65, and 43 differentially expressed AS events (DEASE) were identified in HT_1h vs Con, HT_72h vs Con, and Sen vs Con, respectively (Fig. 3b).

### Analysis of differentially expressed genes and isoforms during HS and senescence

**Fig. 4:**
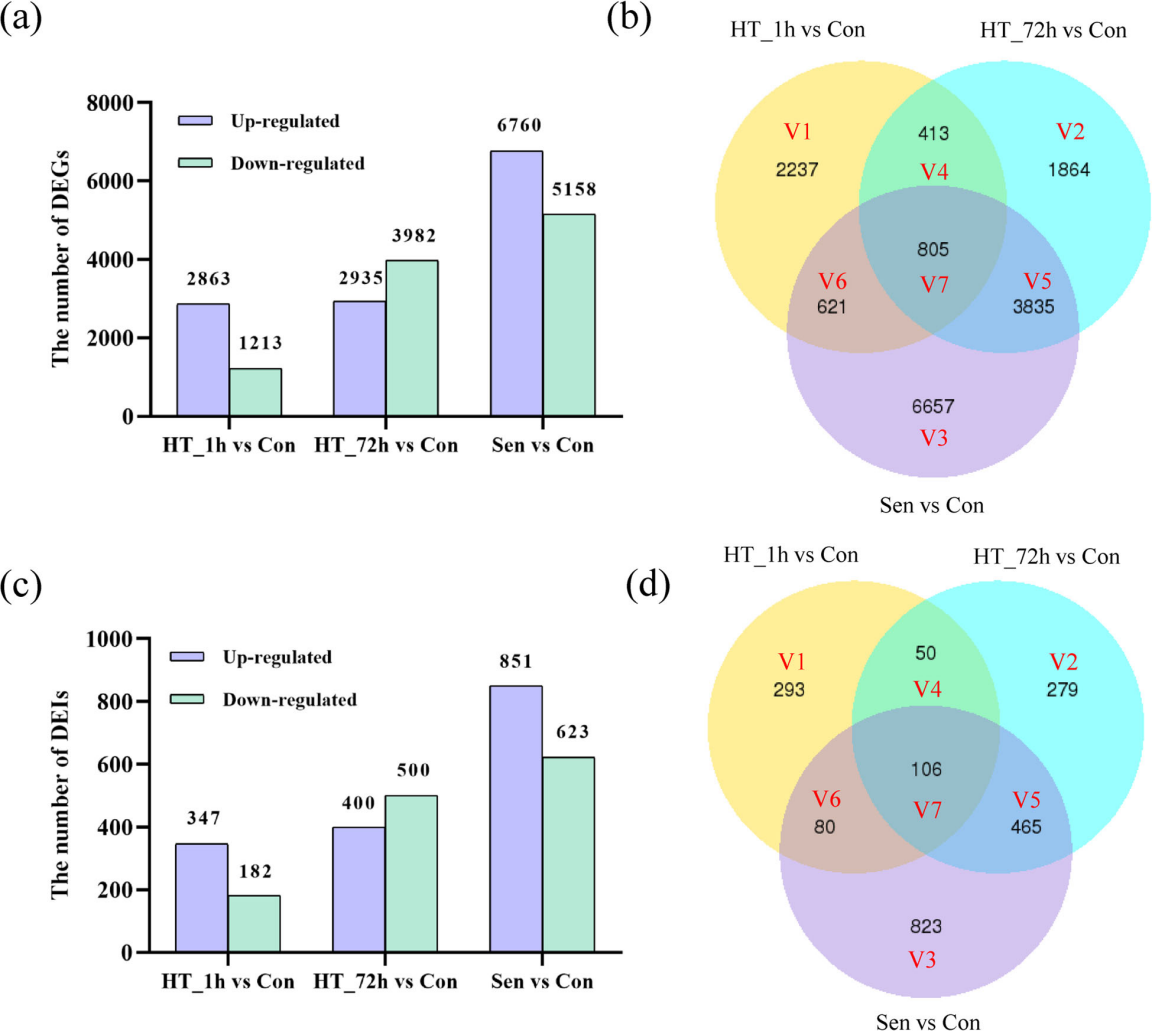
Analysis of differentially expressed genes (DEGs) and isoforms (DEIs) in response to heat stress and natural senescence. **a** The number of up- and down-regulated DEGs at each time point of heat stress and senescence stage. **b** Venn diagram of DEGs in three comparisons. **c** The number of up- and down-regulated DEIs at each time point of heat stress and senescence stage. **d** Venn diagram of DEIs in three comparisons. V1, only HT_1h vs Con; V2, only HT_72h vs Con; V3, only Sen vs Con; V4, the intersection of HT_1h vs Con and HT_72h vs Con but Sen vs Con; V5, the intersection of HT_72h vs Con and Sen vs Con but HT_1h vs Con; V6, the intersection of HT_1h vs Con and Sen vs Con but HT_72h vs Con; V7, the intersection of HT_72h vs Con, Sen vs Con and HT_1h vs Con

To investigate the gene expression pattern under HS or natural senescence, DEGs were identified in three comparisons, including HT_1h vs Con, HT_72h vs Con, and Sen vs Con, with at least a two-fold difference and a *p*-value less than 0.05 (|log_2_ Ratio (Treatment/Control)| ≥ 1, *p* ≤ 0.05). To reduce noise and improve the reliability of DEG analysis, the genes with below 10 FPKM in both control and treatment databases were all removed. As a result (Fig. 4a), a total of 4076 (2863 up-regulated genes and 1213 down-regulated genes), 6917 (2935 up-regulated genes and 3982 down-regulated genes), and 11,918 (6760 up-regulated genes and 5158 down-regulated genes) DEGs were detected in HT_1h vs Con, HT_72h vs Con, and Sen vs Con, respectively. Further analysis using a Venn diagram (Fig. 4b) showed that both unique and overlapping sets of DEGs were detected at each comparison. For the comparison of HT_72h vs Con, most DEGs (3835; 55.44%) belonged to V5 (the intersection of HT_72h vs Con and Sen vs Con but HT_1h vs Con). However, most DEGs (2237; 54.88%) in the comparison of HT_1h vs Con were unique to HT_1h.

It has been known that post-transcriptional regulation, including alternative splicing can also change the expression of transcripts. Therefore, differentially expressed isoforms (DEIs, |log_2_ Ratio (Treatment/Control)| ≥ 1, *p*-value ≤0.05, FPKM ≥10) produced by AS were also identified during heat stress and leaf senescence (Fig. 4c). Similar to DEGs, most DEIs (293/529, 55.39%) which were induced by HT_1h were responsive to short-time heat stress specially, whereas most DEIs (465/900, 51.67%) which were induced by HT_72h also responded to leaf senescence (Fig. 4d).

### Functional analysis of DEGs and DEIs co-regulated by short- and long-term HS

**Fig. 5:**
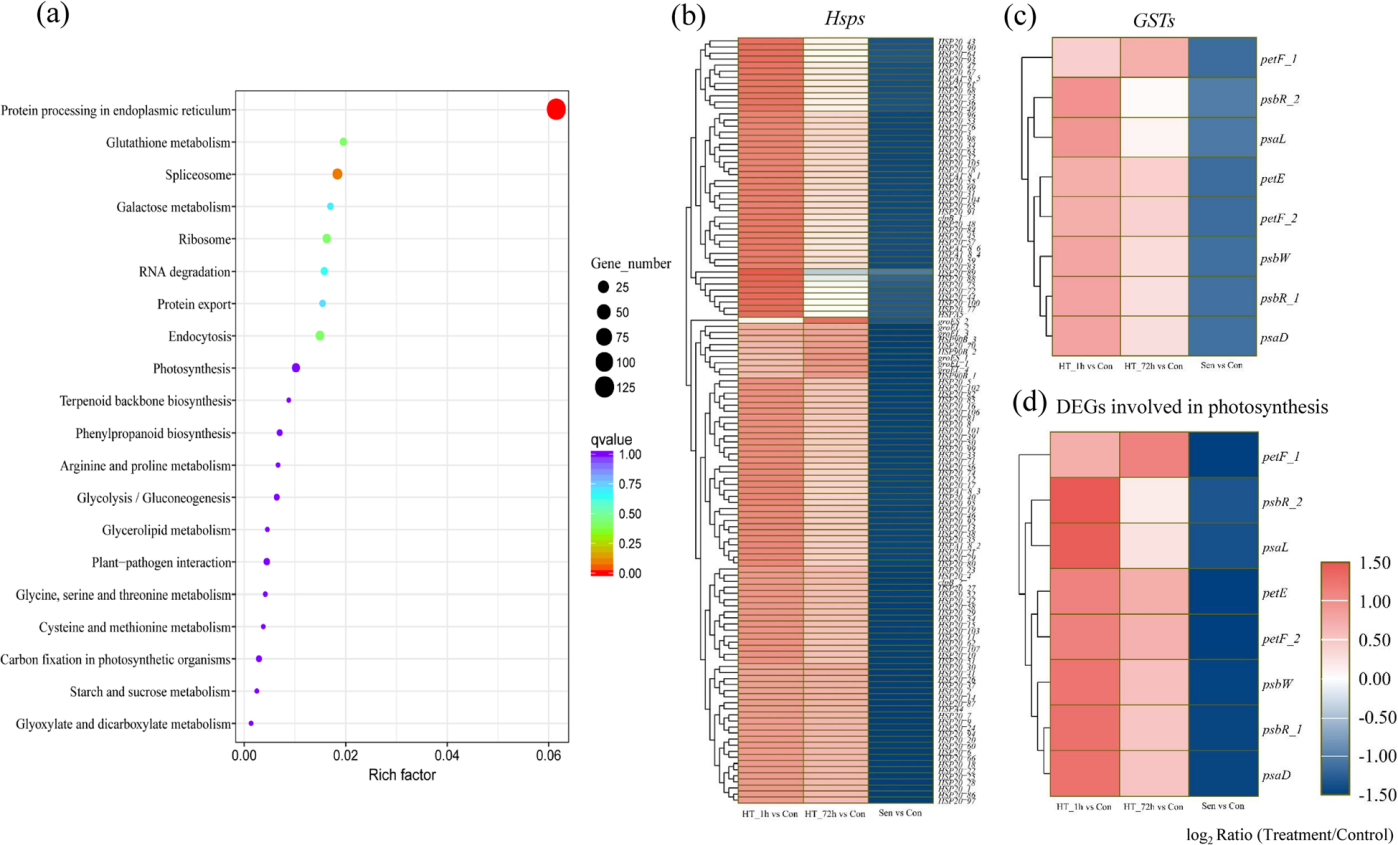
Analysis of DEGs co-induced by HT_1h and HT_72h. **a** The top 20 KEGG pathways of up-regulated genes co-induced by short- and long-term heat treatments. **b**-**d** Hierarchical clustering analysis of *Hsps* (b), *GSTs* (**c**), and DEGs involved in photosynthesis (**d**) co-induced by short- and long-term heat stress during heat stress and senescence. The difference in expression is represented by a color block, and the color of the block ranges from blue (down-regulated) to red (up-regulated). *Hsp*, *heat shock protein*; *GST*, *Glutathione S-transferase*

There were 413 DEGs in DEG_V4 (the intersection of comparison HT_1h vs Con and HT_72h vs Con but Sen vs Con), including 289 persistently up-regulated genes and 101 persistently down-regulated genes. Detailed metabolic pathway analysis (Fig. 5a) showed that most of these up-regulated genes were significantly involved in protein processing in endoplasmic reticulum (125 DEGs), and spliceosome (17 DEGs). Further analysis found that the up-regulated genes involved in the above pathways mainly encoded Hsps, including Hsp20, HspA5, Hsp90A, Hsp90B, and HspA1_8. They accounted for 43.60% (126/289) of the up-regulated DEGs. Heatmap (Fig. 5b, Additional file 3) of all induced *Hsps* showed that *Hsps* were strongly induced by HS, and the expressions of *Hsp* genes under short-term heat treatment were always higher than those under long-term heat treatment. Glutathione S-transferases (GSTs) are known to play a key role in the detoxification and reduction of ROS. A total of five genes encoding GSTs were up-regulated by both short- and long-term HS (Fig. 5c, Additional file 3). In addition, eight genes involved in photosynthesis, including *psaD*, *psaL*, *psbC*, *psbRs*, *psbW*, *petE*, and *petF* were responsive both to short- and long-term HS (Fig. 5d, Additional file 3). Among the 101 down-regulated genes, only one *Hsp* gene (*i0_LQ_TF3rd_c75113/f1p27/835*) was identified.

For DEI analysis, a total of 50 DEIs, including 28 persistently up-regulated isoforms and 17 persistently down-regulated isoforms were identified in DEI_V4 (the intersection of comparison HT_1h vs Con and HT_72h vs Con but Sen vs Con). Similar to DEGs in DEG_V4, half of up-regulated DEIs encoded Hsps.

### Short- and long-term HSR genes and isoforms encoded distinct functional groups

**Fig. 6:**
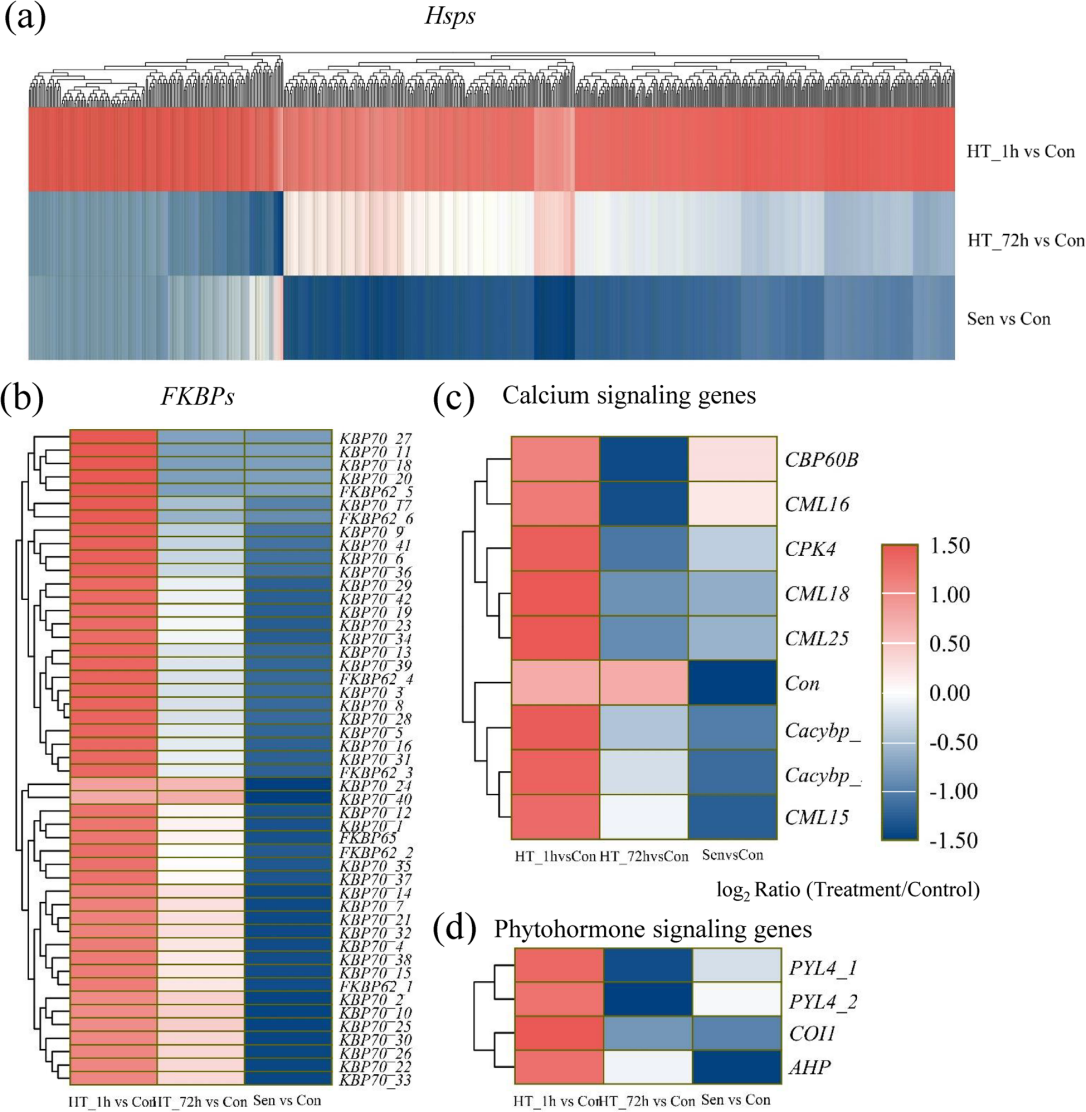
Heatmaps of DEGs specifically induced by HT_1h. **a**-**d** Hierarchical clustering analysis of *Hsps* (**a**), *FKBPs* (**b**), calcium signaling genes (**c**), and phytohormone signaling genes (**d**) specifically up-regulated by HT_1h during heat stress and senescence. The difference in expression is represented by a color block, and the color of the block ranges from blue (down-regulated) to red (up-regulated). *FKBP*, *FK506-binding proteins*

Although there was an overlap, a set of genes exhibited altered expression patterns specific to short- or long-term HS. A total of 2237 genes and 293 isoforms were specifically responsive to HT_1h, most of which were upregulated (2126/2237; 265/293). Among the up-regulated genes and isoforms, 1626 DEGs and 193 DEIs exhibited at least 32-fold changes. The KEGG analysis showed that genes specifically induced by HT_1h were significantly involved in protein processing in endoplasmic reticulum, endocytosis, plant-pathogen metabolism, spliceosome, and protein export (Additional file 4a). DEGs enriched in these pathways almost all encoded Hsps. A total of 658 differentially expressed *Hsp* genes were identified, accounting for 30.95% (Fig. 6a, Additional file 5). FK506-binding proteins (FKBPs), peptidyl-prolyl cis/trans isomerases, which have been reported to interact with HSP90 were also specifically induced by short-term heat stress (Fig. 6b, Additional file 5) [30]. After 1 h of heat treatment, six *FKBP62*, one *FKBP65* and 42 *FKBP70s* were significantly up-regulated. In addition, several Ca^2+^ signaling genes were also significantly up-regulated after 1 h of heat treatment, including one *Calcium-Dependent Protein Kinase 4* (*CPK4*), two *Calcyclin Binding Proteins* (*Cacybps*), one *Calmodulin-Binding Protein 60B* (*CBP60B*), one *Calmodulin-like Protein 15* (*CML15*), one *CML16*, one *CML18*, and one *CML25/26* (Fig. 6c, Additional file 5). Phytohormones play an important role in abiotic stress. Four genes participated in signal transduction of abscisic acid (ABA), jasmonic acid (JA) and cytokinin, including two *PYL4s*, one *Coronatine Insensitive 1* (*COI1*), and one *Histidine Phosphotransfer Protein* (*AHP*) were specifically responsive to short-term HS (Fig. 6d, Additional file 5). Then, the KEGG analysis of DEIs specifically induced by HT_1h was also performed. Protein processing in endoplasmic reticulum, plant-pathogen interaction, cutin, suberine and wax biosynthesis, and spliceosome were significantly enriched (Additional file 4b). The *Hsps* still accounted for the largest part of DEIs; however, the ratio (20.38%, 54/265) was much lower than that of DEGs.

**Fig. 7:**
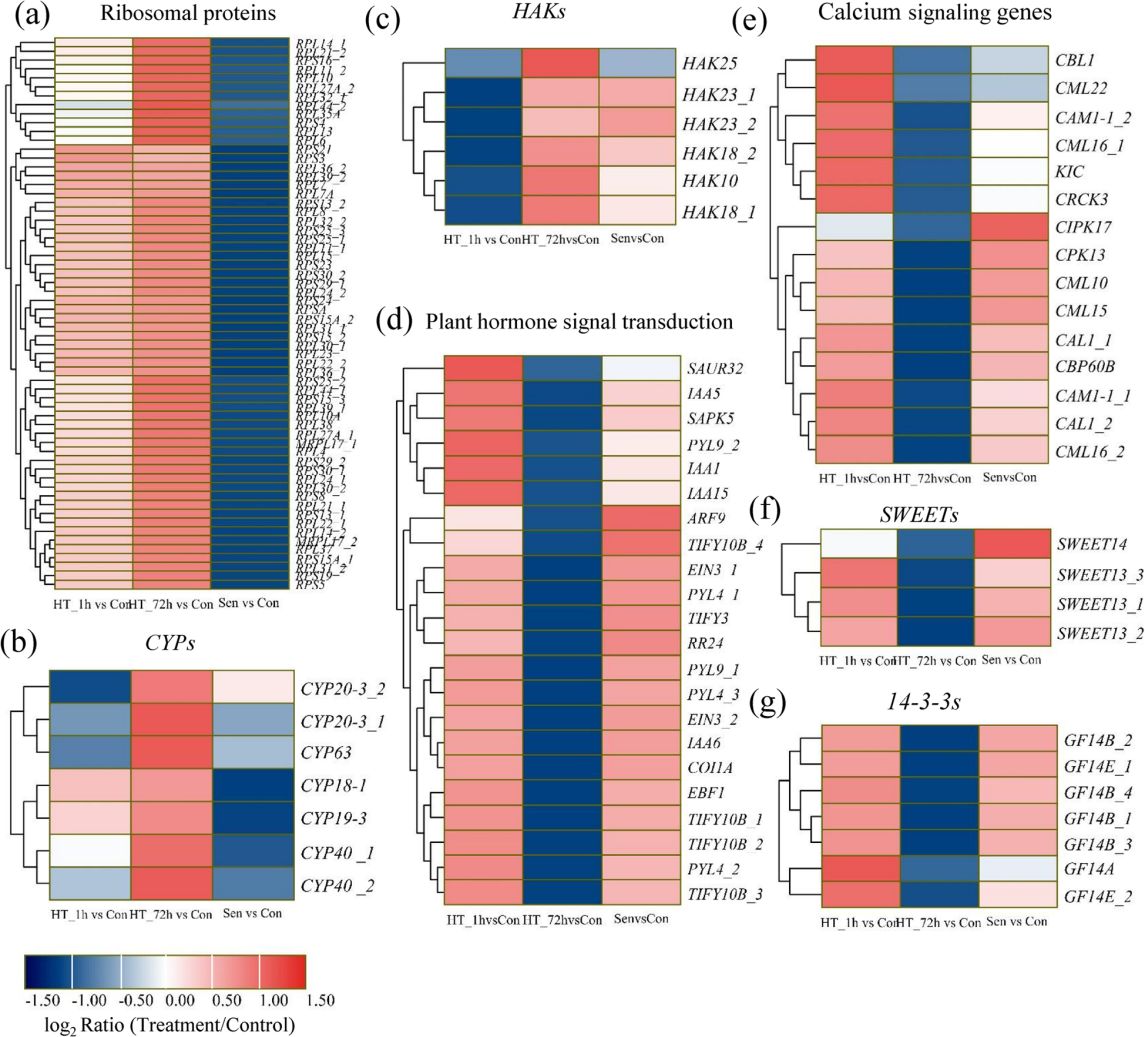
Heatmaps of DEGs specifically regulated by HT_72h. **a**-**c** Hierarchical clustering analysis of ribosomal proteins (**a**), *CYPs* (**b**), and *HAKs* (**c**) specifically up-regulated by HT_72h during heat stress and senescence. **d**-**g** Hierarchical clustering analysis of phytohormone signaling genes (**d**), calcium-related genes (**e**), *SWEETs* (**f**), and *14–3-3 s* (**g**) specifically down-regulated by HT_72h during heat stress and senescence. The difference in expression is represented by a color block, and the color of the block ranges from blue (down-regulated) to red (up-regulated)

After 72 h of high-temperature treatment, a total of 1336 DEGs and 197 DEIs were specially activated. Among the up-regulated DEGs and DEIs, only 104 DEGs and nine DEIs showed more than 32-fold changes, the number of which was much less than that of DEGs specifically induced by HT_1h. Compared with genes induced by HT_1h, the molecular function of DEGs activated by HT_72h was dramatically different. The most enriched pathways were ribosome, ribosome biogenesis in eukaryotes, RNA polymerase, pyrimidine metabolism, and purine metabolism (Additional file 6a). Only seven *Hsps* were specifically induced by HT_72h. A large number of ribosomal protein genes were specifically induced by HT_72h, including 23 *small subunit ribosomal proteins* (*PRSs*) and 39 *large subunit ribosomal proteins* (*PRLs*) (Fig. 7a, Additional file 7). *CYP* genes, also encoding peptidyl-prolyl cis-trans isomerases, were specifically induced by 72 h of heat treatment (Fig. 7b, Additional file 7). In addition, six KT/HAK/KUP transporters, including one *HAK10*, two *HAK18s*, two *HAK23s*, and one *HAK25* were identified in the DEG_V2 (Fig. 7c, Additional file 7). For DEIs specifically induced by HT_72h, no *Hsps* were identified. Differentially spliced *CYPs*, *HAKs*, and *PRLs* were significantly induced by HT_72h as well. Unlike 1 h of heat treatment, relative more DEGs and DEIs were suppressed during 72 h of heat stress. A total of 735 DEGs and 100 DEIs were uniquely down-regulated by HT_72h. The most enriched pathway was plant hormone signal transduction (Additional file 6b). A total of 22 DEGs involved in auxin, cytokinin, ABA, ethylene, and JA signal transduction were identified, such as *Jasmonate ZIM-Domain* (*JAZ*), E*thylene Insensitive 2* (*EIN2*), *Small Auxin Up RNA 32* (*SAUR32*), *etc* (Fig. 7d, Additional file 7). Although several Ca^2+^ signaling genes were induced by HT_1h, Ca^2+^ related genes were mainly down-regulated after 72 h of heat treatment (Fig. 7e, Additional file 7). In addition, sugar transporters, including three *SWEET13s* and one *SWEET4*, were specially suppressed by HT_72h (Fig. 7f, Additional file 7). Interestingly, seven 14–3-3 proteins (one *GF14A*, four *GF14Bs*, and two *GF14Es*) were identified as DEGs uniquely down-regulated after 72 h of heat treatment (Fig. 7g, Additional file 7). In terms of DEIs specifically suppressed by HT_72h, several DEIs involved in plant hormone signal transduction and Ca^2+^ signaling were generated by alternative splicing.

### Analysis of DEGs and DEIs co-regulated by senescence and HS

**Fig. 8:**
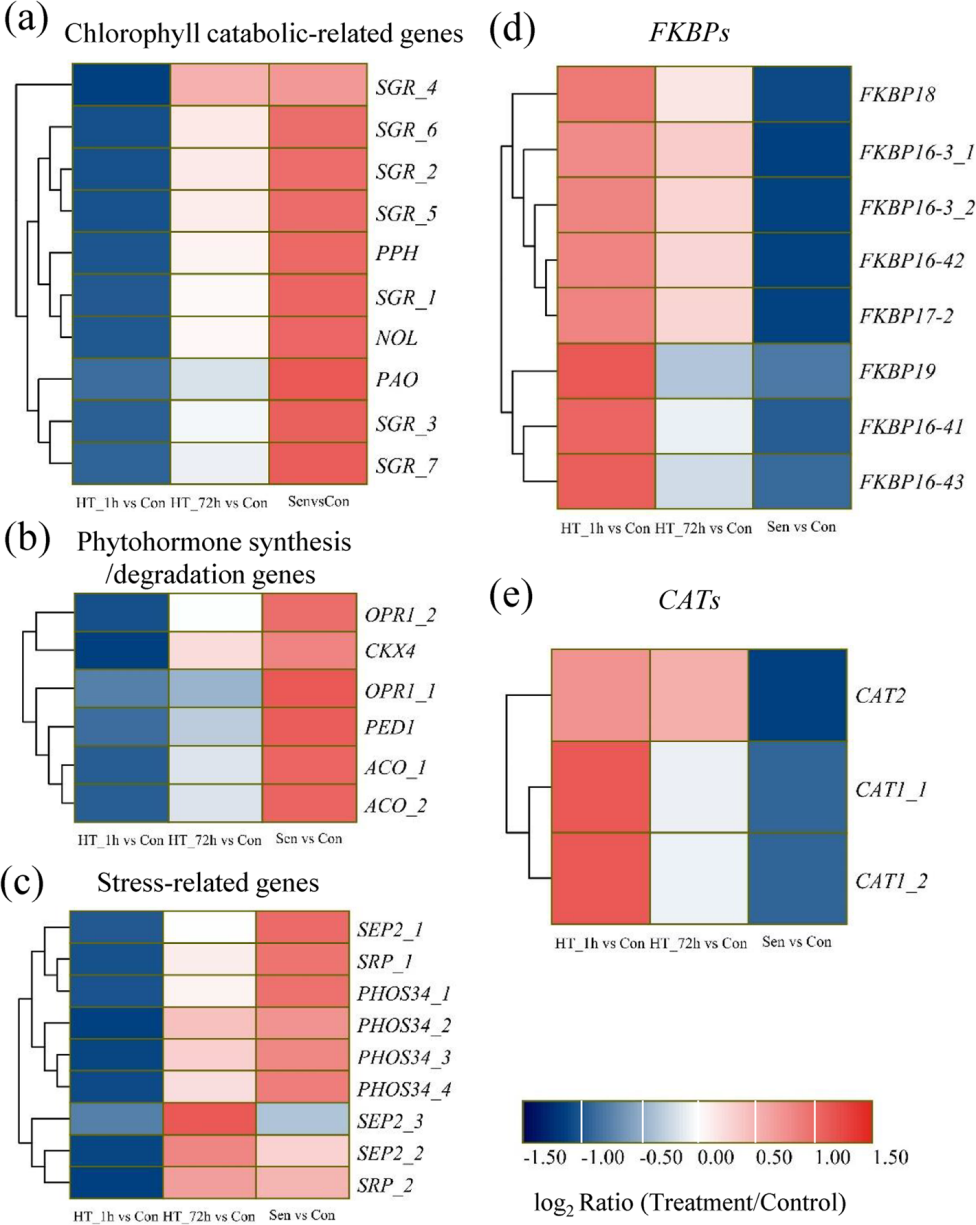
Heatmaps of DEGs co-regulated by HT_72h and Sen. **a**-**c** Hierarchical clustering analysis of chlorophyll catabolic-related genes (a), phytohormone synthesis/degradation genes (**b**), and stress-related genes (**c**) co-induced by HT_72h and Sen during heat stress and senescence. **d**-**g** Hierarchical clustering analysis of *FKBPs* (d), and *CATs* (e) co-suppressed by HT_72h and Sen during heat stress and senescence. The difference in expression is represented by a color block, and the color of the block ranges from blue (down-regulated) to red (up-regulated). CAT, catalase

As previously reported, the sustained high temperatures could accelerate leaf senescence [18]. Here, we found that most of the genes (55.44%, 3835/6917) and isoforms (51.67%, 465/900) responsive to HT_72h also responded to Sen (Fig. 4b). A total of 1193 genes and 164 isoforms were induced commonly by HT_72h and Sen. The marker gene of leaf senescence *SAG12* was significantly induced by 72 h of heat stress and natural senescence. In addition, this up-regulated DEGs list contained four kinds of chlorophyll catabolic-related genes (Fig. 8a, Additional file 8), including seven *staygreens* (*SGRs*), one *pheophorbide a oxygenase* (*PAO*), one *pheophytinase* (*PPH*), and one *chlorophyll b reductase* (*NOL*). Among these up-regulated DEGs, five ethylene and JA biosynthesis genes, and one cytokinin oxidase gene were identified (Fig. 8b, Additional file 8), including one *1-aminocyclopropane-1-carboxylate oxidase* (*ACO*), two *12-oxophytodienoate reductase 1 s* (*OPR1s*), one *peroxisome defective 1* (*PED1*), and one *cytokinin oxidase/dehydrogenase 4* (*CKX4*). In addition, stress-related genes were also identified, including three *stress enhanced protein 2 s* (*SEP2s*), two *stress-related proteins* (*SRPs*), and four universal stress proteins *PHOS34s* (Fig. 8c, Additional file 8). The annotation analysis of DEIs showed that one *PAO*, two *SGRs* and one *PED1* were generated by AS.

The number of down-regulated genes (2546) and isoforms (299) were much more than those of up-regulated genes and isoforms in V5. KEGG analysis showed that the most three enriched pathways were photosynthesis-antenna proteins (176 DEGs), photosynthesis (205 DEGs), and carbon fixation in photosynthetic organisms (147 DEGs) (Additional file 9). DEGs involved in these pathways included a large number of *LHCA*, *LHCB*, *psbA*, *psbP*, *psbW*, *psbR*, *rbcS*, etc. Interestingly, *FKBPs* which were specifically induced by HT_1h were suppressed by HT_72h and Sen (Fig. 8d, Additional file 8). In addition, two *Catalase 1* s (*CAT1s*) and one *CAT2* were down-regulated (Fig. 8e, Additional file 8). The DEI annotation results showed that 18, 11, and 8 DEIs involved in carbon fixation in photosynthetic organisms, photosynthesis-antenna proteins, and photosynthesis were generated by AS, respectively.

It is worth noting that among the 621 genes in V6 (the intersection of HT_1h vs Con and Sen vs Con but HT_72h vs Con), 93 DEGs showed the opposite variation tendency between HT_1h vs Con and Sen vs Con, indicating that there was an antagonistic relationship between senescence and short-term HS. A total of 26 genes were significantly induced by HT_1h, but were suppressed by Sen. On the contrary, a total of 67 genes were significantly repressed by HT_1h, but were induced by Sen. Functional annotations showed that the heat-induced 26 DEGs contained three *Hsps*, three *psbRs*, one *psbP*, one *GST*, and one *Uxin/Indole-3-Acetic Aci*d 3 (*IAA3*). The 67 senescence-induced DEGs contained two *NRT1/PTR Family 6.4 s* (*NPF6.4 s*), two *NDR1/HIN1-like 10s* (*NHL10s*), and one *STP1*. Interestingly, *SAG12*, the most widely used senescence-associated reference gene for characterizing leaf senescence, was observed in the repressed genes by HT_1h.

### Functional analysis of DEGs and DEIs specifically responsive to natural senescence

Besides, genes and isoforms specifically responsive to natural senescence were also investigated. A total of 5119 and 633 up-regulated DEGs and DEIs were identified, respectively. KEGG analysis showed that these up-regulated DEGs were enriched in 108 pathways. The most significantly enriched pathways were valine, leucine and isoleucine degradation, fatty acid degradation, and galactose metabolism (Additional file 10a). Among 108 pathways, 92 were the same as those responsive both to HT_72h and Sen. For DEIs, some senescence-associated genes, including three *early flowering 3 s* (*ELF3s*), one *PAO*, and one abscisic aldehyde oxidase 3 (*AAO3*) were generated by AS.

However, unlike with DEGs responsive both to HT_72h and Sen, much less down-regulated than up-regulated DEGs were identified specifically by Sen. Although many down-regulated DEGs were still enriched in photosynthesis, carbon fixation in photosynthetic organisms, and glyoxylate and dicarboxylate metabolism, most down-regulated DEGs were enriched in the ribosome (Additional file 10b).

### Major transcription factors (TFs) in response to HS and senescence

During HS and senescence, a series of TF families were identified. The major TF families presented were Hsf, and NAC families (Additional file 11, Additional file 12). *Hsfs* were highly responsive to short-term HS. A total of 70 *Hsf*s were specifically induced by HT_1h. In addition, one *Hsf* was specifically up-regulated by HT_72h, four *Hsfs* were up-regulated by both HT_1h and HT_72h, and two *Hsfs* were up-regulated by both HT_72 and Sen. For the four *Hsfs* induced both by HT_1h and HT_72h, the expression abundance of HT_1h was much higher than that of HT_72h. Most of the induced *Hsfs* belonged to class A, including *HsfA2s* and *HsfA6s*. However, only one class C *Hsf* was identified, and it was suppressed specifically by senescence. As shown in Additional file 12, *NACs* were mainly induced by senescence. Totally, 38 *NACs* were specifically induced by Sen, and 16 *NACs* were co-induced by HT_72h and Sen. Only one *NAC* gene was up-regulated by HT_1h specifically. In contrast, down-regulated *NAC* genes were mainly identified under heat stress.

The subsequent AS analysis showed that *Hsfs* were only alternatively spliced during short-term heat stress, including six *HsfA2as*, one *HsfA2d*, one *HsfA2e*, two *HsfB2as*, two *HsfB2bs*, one *HsfB2c*, and eight *HsfA6as*. However, NAC genes were alternatively spliced not only during senescence but also during long-term heat stress. For example, isoforms *i2_LQ_TF3rd_c10489/f1p2/2143* (*NAC071*) and *i2_LQ_TF3rd_c8329/f1p0/2237* (*NAC074*) were generated by alternative splicing in Sen. Isoforms *i1_LQ_TF3rd_c49009/f1p0/1737* (*NAC068*) and *i1_HQ_TF3rd_c9464/f2p0/1360* (*NAC079*) were generated by alternative splicing in Sen and HT_72h.

**Fig. 9:**
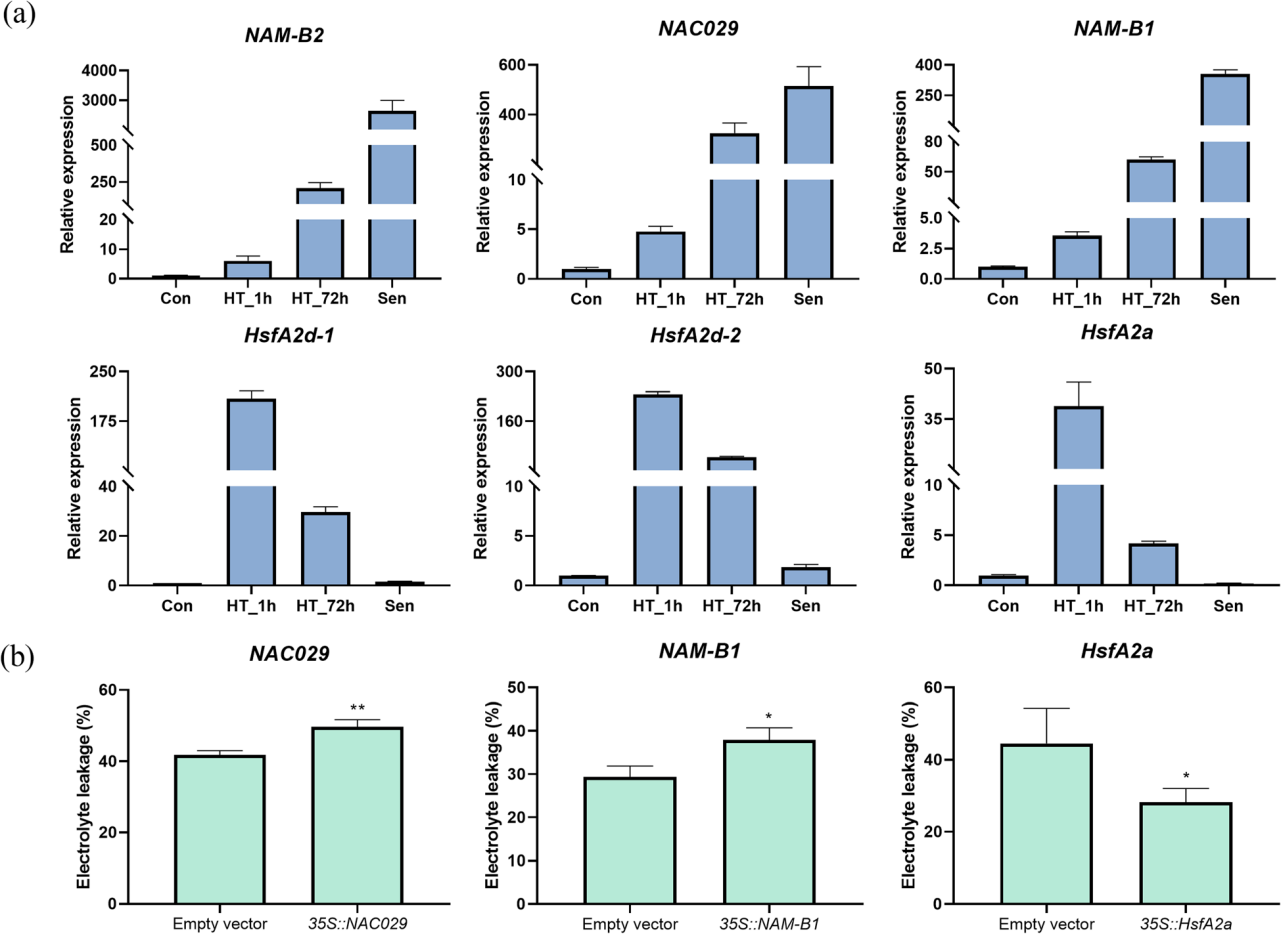
Quantitative real time PCR (qRT-PCR) validation and function analysis of differentially expressed transcription factors. **a** Relative expression of *NAM-B2* (*i1_LQ_TF3rd_c8869/f1p0/1379*), *NAC029* (*i1_HQ_TF3rd_c26766/f2p2/1431*), *NAM-B1* (*i1_HQ_TF3rd_c44692/f2p0/1636*), *HsfA2d-1* (*i1_LQ_TF3rd_c31016/f1p0/1406*), *HsfA2d-2* (*i1_LQ_TF3rd_c80591/f1p0/1429*), and *HsfA2a* (*i3_HQ_TF3rd_c12860/f2p0/3466*). **b** Membrane damages in agro-infiltrated *Nicotiana. benthamiana* plants with *NAC029* (*i1_HQ_TF3rd_c26766/f2p2/1431*), *NAM-B1* (*i1_HQ_TF3rd_c44692/f2p0/1636*), and *HsfA2a* (*i3_HQ_TF3rd_c12860/f2p0/3466*) after 1 day at 45 °C by electrolyte leakage assay compared with agro-infiltrated plants with empty vector. Asterisks indicated significant difference with wild type at high temperature as estimated using Student’s t-test (* *P* < 0.05, ** P < 0.01). Error bars represent the standard error of the mean (*n* = 3)

To validate the results of the RNA sequencing, six DEGs, including 3 *NACs* and 3 *Hsfs*, were selected for quantitative real-time PCR (qRT-PCR) (Fig. 9a). DEGs *i1_LQ_TF3rd_c8869/f1p0/1379* (*NAM-B2*), *i1_HQ_TF3rd_c26766/f2p2/1431* (*NAC029*), and *i1_HQ_TF3rd_c44692/f2p0/1636* (*NAM-B1*) were induced both by Sen and HT_72h. DEGs *i1_LQ_TF3rd_c31016/f1p0/1406* (*HsfA2d-1*) and *i1_LQ_TF3rd_c80591/f1p0/1429* (*HsfA2d-2*) were induced both by HT_1h and HT_72h. DEG *i3_HQ_TF3rd_c12860/f2p0/3466* annotated as *HsfA2a* was specifically induced by HT_1h. As shown in Fig. 9a, the qRT-PCR results of all six DEGs were consistent with those obtained by RNA-seq, indicating that RNA-seq results were reliable.

### Transient expression of two *NACs* and one *Hsfs* in *N. benthamiana* leaves

The function of three TFs, including NAC029, NAM-B1 and HsfA2a, were characterized in transient expression experiments conducted in *N. benthamiana*. The left half of the leaf was infiltrated with an empty vector, and the right half of the same leaf was infiltrated with a binary construct containing 35S promoter and the coding sequence of each TF. After 24 h of heat treatment with 45 °C, cell membrane injury of each sample was measured by electrolyte leakage. As the results show (Fig. 9b), plants agro-infiltrated with the *35S::NAC029* or *35S::NAM-B1* constructs both exhibited significantly higher levels of cell membrane injury compared with controls, whereas plants agro-infiltrated with the *35S::HsfA2a* construct exhibited significantly lower levels of cell membrane injury compared with controls.

## Discussion

HS is the key determinant of growth and development for the cool-season grass tall fescue [2, 3]. High temperatures can result in significant changes in the transcriptome, proteome, and metabolome. A comprehensive understanding of genome-wide HSR genes is the first and essential step to investigate the transcriptional regulatory network of HS response in tall fescue. In this study, SMRT sequencing combined with Illumina sequencing were firstly used in tall fescue. The SMRT sequencing showed a great improvement in mean length of unigenes and the ratio of full-length transcripts compared with the second-generation RNA sequencing [13]. A total of 62,443 unigenes with an average length of 2595 bp in tall fescue were obtained here (Fig. 2). The high ratio of full-length transcripts contributes to further cloning of heat-responsive genes, as well as functional analysis.

### Common and distinct heat-responsive genes in short- and long-term heat stress

In this study, tall fescue plants were treated by short- (1 h) and long-term (72 h) heat stress, respectively. Compared with previous studies in tall fescue (2 h and 12 h) and wheat (1 h and 24 h) [15, 31], a relative small part of genes was co-regulated by short and prolonged heat treatments here. It could be the result of a long interval of two heat treatments. These commonly up-regulated DEGs mainly included *Hsps*, *GSTs*, and photosynthesis-related genes. Hsps play a crucial role in protecting plants against HS by re-establishing normal protein conformation and thus cellular homeostasis [5]. Although the induction of *Hsps* during heat stress has been extensively reported [7, 32, 33], *Hsps* were just a fraction of heat-induced transcripts in previous studies [13, 34, 35]. There were only 7.75% (117/1509) of DEGs annotated as *Hsps* in wheat after 1 h of HS [31, 34]. Inconsistently, the *Hsp*s made the largest proportion, almost half of the commonly up-regulated DEGs in tall fescue after heat treatments. In addition, the expression of these *Hsps* were altered more dramatically in HT_1h than in HT_72h, which is consistent with the previous research [31]. The result indicates that the response of *Hsps* to heat stress is extensive and quite fast. Heat stress produces ROS which is toxic to plant cell and affects the repair system of photosystem II [36, 37]. Several studies demonstrated that ROS-scavenging mechanisms have an important role in protecting plants against high-temperature stress [38]. In this study, ROS scavengers (*GSTs*) and genes involved in photosynthesis were both co-induced by HT_1h and HT_72h, implying that tall fescue plants try to scavenge ROS and maintain the steady photosynthesis to minimize the damages caused by high temperature.

Heat responses of tall fescue were distinct in terms of heat treatment time. Here, we found that the short-term heat response in tall fescue is a much quicker and a more dramatic process than ever reported. In the previous study, the number of DEGs under 2 h of short-term heat treatment (4311) was only half of that under 12 h of long-term heat treatment (8395), and most of the DEGs were down-regulated [15]. However, in our study, much more DEGs were specifically regulated by HT_1h, and 76.48% of which were up-regulated more than 32 fold. The much quicker response of short-term HS was further highlighted by a study that showed that 42.83 and 74.50% DEGs in leaves and grain of wheat were detected within the first 30 min of heat treatment [39]. After 1 h of heat stress, half of the DEGs were *Hsps*; however, almost no *Hsps* were up-regulated specifically by HT_72 h, further indicating that the response of *Hsps* to heat stress is fast, short and drastic in tall fescue. *FKBPs*, another chaperone family with the classical role in protein folding [40], were also highly induced by HT_1h. Inconsistency with that *FaFKBP65* was induced from 0 to 13.33 by HT_1h in this study, the expression of *OsFKBP65* was detected at the RNA level only after heat stress [41]. In *Arabidopsis*, AtFKBP62 modulates thermotolerance by interacting with Hsp90.1 and affecting the accumulation of HsfA2-regulated sHsps [30]. Here, six *FaFKBP62s* were significantly induced by HT_1h. The results suggest that *FKBPs* are activated to resist short-term high temperature in tall fescue. Calmodulin has been reported to be involved in FKBP functioning, and the role of calcium signaling in HSR has been demonstrated in wheat and *arabidopsis* [7, 42, 43]. Consistently, *PCM5* which encodes calmodulin was up-regulated by HT_1h. Besides *PCM5*, other calcium signaling genes were also identified after 1 h of heat treatment, suggesting a possible role of Ca^2+^ mediated signaling in short-term HSR. The roles of plant hormones, such as ABA, SA, and ethylene in thermotolerance have been well reported [9, 44]. Here, genes involved in ABA, brassinosteroids, JA, and cytokinin were induced by short-term HS, although the roles these up-regulated genes in heat stress have not been demonstrated. Additional experiments are needed to determine their roles and downstream pathways in thermotolerance.

The process of long-term HSR was quite different from that of short-term HSR. Compared with short-term HSR, more genes were suppressed. After long-term heat stress, ribosomal proteins, *CYPs*, and potassium transporters were significantly induced, while phytohormone signaling genes, calcium signaling genes, *SWEETs*, and *14–3-3 s* were suppressed. Ribosomal proteins accounted for the largest proportion of DEGs that were up-regulated specifically by HT_72h. Consistently, a large number of ribosomal protein genes were up-regulated after HS, especially the long-term HS in wheat [31]. Given that ribosomal proteins are essential for protein synthesis, the induction of ribosomal proteins may indicate their role in preventing interference of protein synthesis by long-term heat stress. Potassium ion (K^+^) is an essential nutrient for life, and K^+^ transport across membranes is mediated by the KT/HAK/KUP family [45]. *KT/HAK/KUP* transporters have been documented to participate in stress responses, including salt and drought [46, 47]. However, the role of *KT/HAK/KUP* transporters in heat stress has not been reported. Here, the upregulation of *HAKs* supplies a clue for further investigation of HAK-mediated heat response. They may rescue cellular osmotic imbalance caused by high temperature through K^+^ uptake and efflux. In contrast to HT_1h, genes involved in plant hormone signal transduction, including auxin, cytokinin, ABA, ethylene, and JA were mainly suppressed by HT_72h. JA and ethylene were reported to have opposite roles in heat stress tolerance in *Arabidopsis*. The *coi1* mutant is more susceptible to heat stress, whereas the *ein2* mutant shows increased thermotolerance [48, 49]. However, the expressions of *FaCOI1* and *FaEIN2* were both reduced after HT_72h, indicating that the roles of *COI1* and *EIN2* in heat stress may be species-specific. 14–3-3 proteins function as regulators in various abiotic and biotic stresses [50]. In *Drosophila* cells, a 14–3-3 protein was observed to dissolve and re-naturalize thermal-aggregated proteins [51]. However, the function of 14–3-3 protein in HS has not been reported in plants. Here, we found that long-term HS was associated with 14–3-3 proteins in tall fescue. Several *GF14s* were suppressed specifically by HT_72h. In contrast, most of the 14–3-3 proteins showed a transient elevation of expression after 15 min of heat treatment in *Arabidopsis* [52].

### The synergetic and antagonistic relationships between heat stress and senescence

The relationship between senescence and short- or long-term heat stress was also investigated in this study. There may be an antagonistic relationship between short-term HS and leaf senescence; however, short-term heat stress could accelerate leaf senescence. *SAG12*, a reference gene for leaf senescence, increases remarkably with age [53]. It was decreased by HT_1h but increased by HT_72h, indicating that leaf senescence was somewhat reduced after short-term HS, but induced after long-term HS. In previous studies, heat-induced leaf senescence was negatively associated with cytokinin synthesis, and positively associated with ethylene and ABA [18, 19]. Consistently, ethylene synthesis gene *ACO* and cytokinin degradation gene *CKX4* were induced by both HT_72h and Sen. In addition, synthesis genes of another senescence-promoting phytohormone JA were also induced by HT_72h and Sen. Our results indicated that long-term heat stress could promote senescence by regulating genes involved in phytohormone synthesis and degradation. During leaf senescence, chlorophyll is degraded to colorless products via SGR and chlorophyll catabolic enzymes (CCEs), such as NYC1, NOL, PPH, PAO, and RCCR [54–56]. SGR-CCE-LHCII complexes are formed to trigger LHCPII destabilization for the degradation of Chls and Chl-free LHCPII subunits in senescing leaves [57]. Here, SGR and three CCEs were induced but LHCbs were suppressed by both HT_72h and Sen, indicating that long-term heat stress accelerates senescence through SGR-CCE-LHCII complexes.

### The responses of *FaHsfs* and *FaNACs* to heat stress and leaf senescence

TFs play pivotal roles in response to diverse abiotic and biotic stresses. Here, members of heat-responsive TF families were identified. Hsfs are the master regulators of the HSR in all eukaryotic organisms [58]. Here, expression levels of *HsfA2s* were the most strongly induced by HS, which was consistent with previous studies [12, 59]. Several studies have reported function analysis of *HsfA2*, but it is still under controversy [10, 60, 61]. In this study, *N. benthamiana* transiently overexpressing *FaHsfA2a* displayed increased tolerance to HS. The further in vivo functional analysis of it will be carried out in tall fescue. Class B Hsfs were always supposed to have repressor functions during HS because of –LFGV-motifs. A small part of *HsfB2s* was induced by short- or long-term HS, and their functions need to be investigated further [61]. Few class C Hsfs have been identified in *Arabidopsis* or tomato, the function of which is still unknown. Consistently, only one *HsfC* was induced by HS in tall fescue. NAC, a specific transcription factor family for plants, plays diverse roles in plant development and abiotic stresses [62]. Several NACs have been identified as positive regulators for senescence, interestingly, some of which are involved in plant stress tolerance as well [63, 64]. Here, many of *NACs* were significantly induced by natural senescence and long-term HS, indicating their potentially important roles in heat-induced senescence. NAC029, a key regulator for natural senescence, has also been identified to be involved in salt, drought, and cold stresses [63, 65–67]. However, the role of NAC029 in HS is still unknown. One tall fescue gene *i1_HQ_TF3rd_c26766/f2p2/1431* which was annotated as *NAC029* was found to be largely induced by both Sen and HT_72h. *N. benthamiana* transiently overexpressing *FaNAC029* displayed increased membrane injury, suggesting that *FaNAC029* may play an important role in senescence accelerated by long-term HS in tall fescue. Meanwhile, functional analysis of another *NAC* gene *i1_HQ_TF3rd_c44692/f2p0/1636* which was annotated as *NAM-B1* was performed in *N. benthamiana* as well. It was reported that *NAM-B1* could accelerate senescence in wheat [68], and post-anthesis heat and a *NAM-B1* introgression have similar (both could accelerate peduncle senescence) but non-additive effects in bread wheat [69]. However, in this study, the leaf infected with *35S::NAM-B1* showed more significant membrane injury after HS compared with that infected with an empty vector, indicating that *FaNAM-B1* may play an important role in senescence accelerated by long-term HS in tall fescue. Further in vivo functional analysis of these two *NAC genes* will be carried out in tall fescue.

### The involvement of AS in heat stress and leaf senescence

AS is involved in many plant processes, especially responses to environmental stresses [70]. In this study, we only detected 298 AS events responsive to HS, the number of which was much less than that in other studies [71]. This may be due to the lack of a reference genome sequence. However, the distribution of AS events in tall fescue is consistent with other plants in which RI is the major AS event [29]. It is well documented that *Hsfs* and *Hsps* are easily alternatively spliced [26, 72]. Differentially expressed *Hsps* and *Hsfs* isoforms generated by AS were widely observed after heat treatment in tall fescue. In addition, many other isoforms, such as *SGR*, *PAO*, and *PED1* produced by AS were also differentially expressed during heat stress in tall fescue. Although no study investigating the role of AS in senescence was reported in plants, *NAC* genes, *ELF3s* and *AAO3* generated by AS were observed in senescent leaves in tall fescue. The function of AS for these genes during HS and senescence needs further investigation.

## Conclusions

**Fig. 10:**
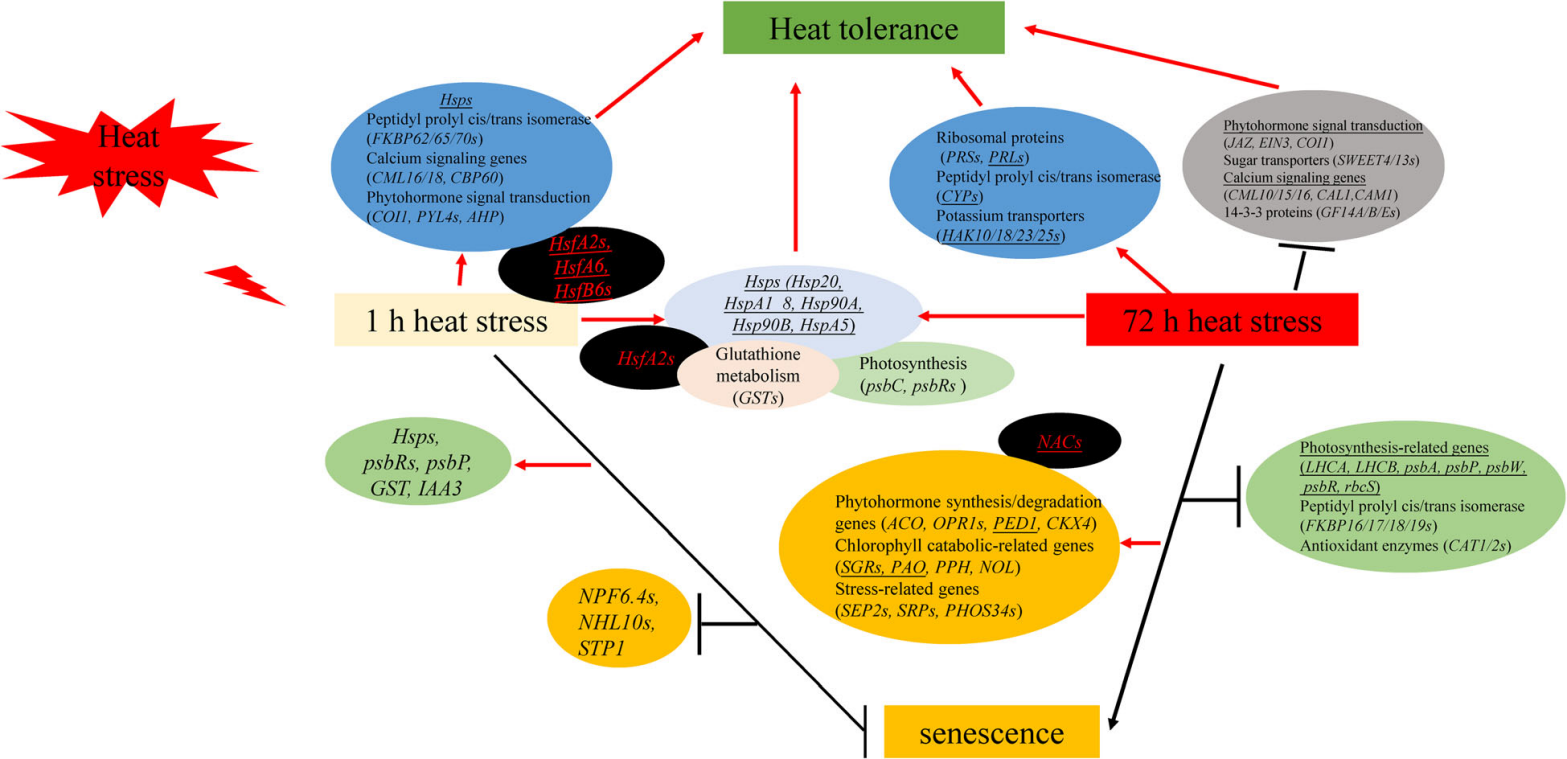
A hypothetical model of heat stress response and the crosstalk between heat stress and leaf senescence in tall fescue. Underlines represent that the differentially expressed isoforms were produced by AS

The short- and long-term HSR and the relationship between HS and senescence were investigated in this study (Fig. 10). Our results showed that the short-term HS can activate a large number of *Hsps*, *FKBPs*, calcium signaling genes, phytohormone signaling genes, and *Hsfs* to improve thermotolerance. However, long-term HS may lead to leaf senescence via activating chlorophyll catabolic genes, phytohormone synthesis/degradation genes, stress-related genes and, NAC TFs, and suppressing photosynthesis-related genes, *FKBPs* and *CATs*. During heat stress and senescence, a large number of HS- and senescence-associated DEIs were generated by AS. Further studies involving in-depth characterizations of heat-resistance breeding candidate genes in tall fescue are required to reveal the underlying mechanism of heat adaptation and heat-induced senescence.

## Methods

### Plants materials and treatments

Seeds of commercial-type tall fescue ‘Houndog 5’ with relatively high thermotolerance (Additional file 13) [73] provided by Baisite Company (Beijing) were sown in plastic pots (10 cm diameter, 10 cm height) with a mixture of sand and peat soil (1/2, v/v). Pots were then maintained in the greenhouse at a temperature of 22 °C/18 °C (day/night), a photoperiod of 14 h light/10 h dark, and relative humidity of 70–80%. After 2 months of establishment, all plants were transferred into controlled-environment growth chambers (HP300GS-C, Ruihua Instrument, Wuhan, China), with a 16 h photoperiod, photosynthetically active radiation at 450 μmol m^− 2^ s^− 1^ at the canopy level, a day/night temperature of 22/18 °C, and 70% relative humidity. The plants acclimated in this condition for 1 week before they were exposed to heat treatments. After one weak, green leaves of tall fescue were collected as control (named as Con). Then the remaining plants were divided into two groups. Group I of tall fescues were kept at 22/18 °C (day/night) in the controlled-environment growth chambers until the leaves of tall fescues appeared senescent, and the senescent leaves were collected (named as Sen). Group II was treated with 38 °C day/33 °C night in the controlled-environment growth chambers. In addition to temperature, other growth environment was as same as control. Leaves were collected after 1 h (named as HT_1h) and 72 h (named as HT_72h) of heat treatments, respectively. All collected samples were immediately frozen in liquid nitrogen and stored at − 80 °C until RNA isolation. All the treatments were repeated for three replications.

### RNA preparation and Illumina RNA sequencing

Total RNA was extracted using the Trizol kit (Invitrogen, Carlsbad, CA) from the collected samples (Con, Sen, HT_1h, and HT_72h). Quantity and quality of the total RNA were detected by NanoDrop 2000 (Thermo, Waltham, USA) and a 1.2% agarose gel. A total of 1.5 μg RNA per sample was used for Illumina RNA sequencing. Sequencing libraries were constructed using NEBNext® Ultra™ RNA Library Prep Kit for Illumina® (NEB, Ipswich, USA) according to the manufacturer’s recommendations. Library quality was evaluated on the Agilent Bioanalyzer 2100 system.

### PacBio SMRT sequencing of pooled RNA samples

For PacBio SMRT sequencing, a pooled RNA sample was mixed from 12 RNA samples used for Illumina RNA-seq with equal amounts. The Isoform Sequencing (Iso-Seq) library was generated following the Iso-Seq protocol using the Clontech SMARTer PCR cDNA Synthesis Kit (Takara, Dalian, China) and the BluePippin Size Selection System protocol which is described by Pacific Biosciences (PN 100–092–800-03).

### PacBio data processing

After PacBio sequencing, raw data were processed using the SMRTlink 6.0 software. CCSs were generated from subreads with passes 2, accuracy > 0.8, and max_length of 15,000. According to whether there were 5′- and 3′-cDNA primers, and whether there was a poly-A tail signal preceding the 3′-primer, a CCS or subread sequence was divided into full length, non-full length, and chimeric reads. Full-length and non-full length fasta data were subsequently inputted into the clustering step, which performs isoform-level clustering by ICE, followed by Arrow polishing finally. To improve the accuracy of consensus, additional nucleotide errors of consensus reads were further corrected using the Illumina sequencing data by LoRDEC [65]. Final transcripts were obtained through removing redundancy in corrected consensus by CD-HIT.

### Full-length unique transcript model reconstruction

The non-redundant transcripts were processed with the Coding GENome reconstruction tool (Cogent v3.1, https://github.com/Magdoll/Cogent) with the parameters: --dun_use_partial. In general, Cogent firstly split the input fasta file into chunks of chunk_size and computed the k-mer profile. Each transcript family was further reconstructed into one or several unique transcript models (referred to as UniTransModels) using a De Bruijn graph method.

### Alternative splicing analysis

Error-corrected non-redundant transcripts (transcripts before Cogent reconstruction) were mapped to UniTransModels using gmap-2017-06-20. Splicing junctions for transcripts mapped to the same UniTransModels were examined, and transcripts with the same splicing junctions were collapsed. Collapsed transcripts with different splicing junctions were identified as transcription isoforms of UniTransModels. AS events were detected with SUPPA (https://github.com/comprna/SUPPA) using default settings. Quantification of the differential expression of AS events were carried out by rMATS [74]. The significant differential expression of AS events were filtered with |dpsi| > 0.1 and *p*-value < 0.05; .

### Differential expression analysis based on Illumina data

Gene expression levels of each sample were calculated by RSEM [75]. DESeq R package (1.10.1) was used for differential expression analysis of two groups. DESeq provided statistical routines for determining differential expression in digital gene expression data using a model based on the negative binomial distribution. The resulting *P* values were adjusted using the Benjamini and Hochberg’s approach for controlling the false discovery rate. A gene was regarded as a DEG if it showed a two-fold difference, |log_2_ Ratio (Treatment/Control)| ≥ 1, the adjusted *p*-value ≤0.05, and FPKM > 10 in either sample of each comparison.

### Quantitative real-time PCR analysis of DEGs

The expression levels of DEGs were performed by qRT-PCR following the previously described method [76]. In brief, cDNA synthesis was performed using a PrimeScript RT reagent kit (Takara, Dalian, China). The qRT-PCR was performed using 20 μl volumes of SYBR (Takara, Dalian, China) and the ABI STEPONE Real-Time PCR system. *FaACTIN* was selected as a reference gene for normalization. All gene-specific primers used for qRT-PCR analysis are listed in Additional file 14. Three independent biological replicates were used for qRT-PCR analysis.

### Transient expression in *N. benthamiana* and electrolyte leakage assay

For the transient overexpression experiments, plasmids were transferred to *Agrobacterium tumefaciens* strain AGL1. Agroinfiltration was performed as described previously [77, 78]. Briefly, overnight-grown bacterial cultures were re-suspended in agro-infiltration medium (10 mM MES, pH 5.6, 10 mM MgCl_2_, and 200 μM acetosyringone) to OD600 of 1.0. Needle-free syringes were used to infiltrate leaves of *N. benthamiana* plants under growing conditions with 24 °C day/20 °C night in a 16 h-light/8 h-dark cycle. After infiltration, the plants were kept at 22 °C overnight. The following morning, the agro-infiltrated plants were moved to 45 °C. After 24 h (in a 12 h-light/12 h-dark cycle) of high-temperature treatment, leaves were harvested for EL assays. Three replicate samples of five 1 cm^2^ leaf discs were punched from each leaf sample and then were washed twice with deionized water. The discs were placed in a 50 mL tube. A total of 25 mL of deionized water was added into each tube, and then samples were kept in a 25 °C shaker with 150 rpm. After 4 h, the initial conductivity was measured using a conductivity meter. Then samples were autoclaved, and total conductivity was determined after cooling to room temperature. The extent of cell membrane injury was calculated as follows: Initial conductivity× 100/total conductivity.

### Measurements of Fv/Fm

Before measuring Fv/Fm, leaves were kept in the dark for 30 min to close all PSII reaction centers. Then, Fv/Fm was measured by a pulse-amplitude modulation (PAM) portable chlorophyll fluorometer (PAM-2500, WALZ, Effeltrich, Germany) according to the manufacturer’s protocol.

## Supplementary information

**Additional file 1.** Numbers and distribution rate of unigenes in seven databases.

**Additional file 2.** Functional annotation of the full-length transcriptome in tall fescue.

**Additional file 3.** The differential expression of DEGs in comparison V4.

**Additional file 4.** KEGG analysis of DEGs and DEIs specifically induced by HT_1h.

**Additional file 5.** The differential expression of DEGs in comparison V1.

**Additional file 6.** KEGG analysis of DEGs specifically regulated by HT_72h.

**Additional file 7.** The differential expression of DEGs in comparison V2.

**Additional file 8.** The differential expression of DEGs in comparison V5.

**Additional file 9.** KEGG analysis of DEGs down-regulated by HT_72h and Sen.

**Additional file 10.** KEGG analysis of DEGs specifically regulated by natural senescence.

**Additional file 11 **The differential expression of *Hsfs* in each comparison.

**Additional file 12.** The differential expression of NAC transcription factors in each comparison.

**Additional file 13.** The phenotypic differences between ‘Houndog 5’ (heat-tolerant) and ‘PI535582’ (heat-sensitive) after heat treatments.

**Additional file 14.** All primers used in this study.

## Data Availability

The datasets generated and analyzed during the current study are available in the NCBI Sequence Read Archive repository under Bioproject PRJNA647166 (https://www.ncbi.nlm.nih.gov/Traces/study/?acc=PRJNA647166).

## Abbreviations

HS: Heat stress
DEG: Differentially expressed gene
Hsp: Heat shock proteins
Hsf: Heat shock factors
ROS: Reactive oxygen species
APX: Ascorbate peroxidase
TF: Transcription factors
SMRT: Single-molecular real-time
AS: Alternative splicing
PacBio: Pacific Biosciences
CCS: Circular consensus sequences
FLNC: Full-length non-chimeric read
ICE: Iterative Clustering for Error Correction
NFL: Non-full-length
Nr: Non-redundant protein sequence database
Nt: Nucleotide sequence database
KOG/COG: Clusters of orthologous groups of proteins
KEGG: Kyoto encyclopedia of genes and genomes
GO: Gene ontology
Con: Control
HT_1h: 1 h of 38 °C heat treatment
HT_72h: 72 h of 38 °C heat treatment
Sen: Senescent leaves
GST: Glutathione S-transferase
ABA: Abscisic acid
JA: Jasmonic acid
SE: Skipping exon
MX: Mutually exclusive exon
A5: Alternative 5′ splice-site
A3: Alternative 3′ splice-site
RI: Retained intron
AF: Alternative first exon
AL: Alternative last exon
DEASE: Differentially expressed AS events
Iso-Seq: Isoform Sequencing
FKBP: FK506-binding Protein
CATs: Catalases
SGR: Staygreen
EL: Electrolyte Leakage
DEI: Differentially Expressed Isoforms
PRS: Small Subunit Ribosomal Protein
PRL: Large Subunit Ribosomal Proteins
PAO: Pheophorbide A Oxygenase
PPH: Pheophytinase
NOL: Chlorophyll b Reductase
ACO: 1-Aminocyclopropane-1-Carboxylate Oxidase
OPR1: 12-Oxophytodienoate Reductase 1
PED1: Peroxisome Defective 1
CKX4: Cytokinin Oxidase/Dehydrogenase 4
SEP2: Stress Enhanced Protein 2
SRP: Stress-Related Proteins
ELF3: Early Flowering 3
AAO3: Abscisic Aldehyde Oxidase 3
HSR: HS Response
CCE: Chlorophyll Catabolic Enzyme
