# Supplementary figures and images for: SMRT and Illumina RNA sequencing reveal novel insights into the heat stress response and crosstalk with leaf senescence in tall fescue
Source: BMC Plant Biol. 2020 Aug 3;20:366. doi: 10.1186/s12870-020-02572-4 (PMC7397585; doi:10.1186/s12870-020-02572-4)

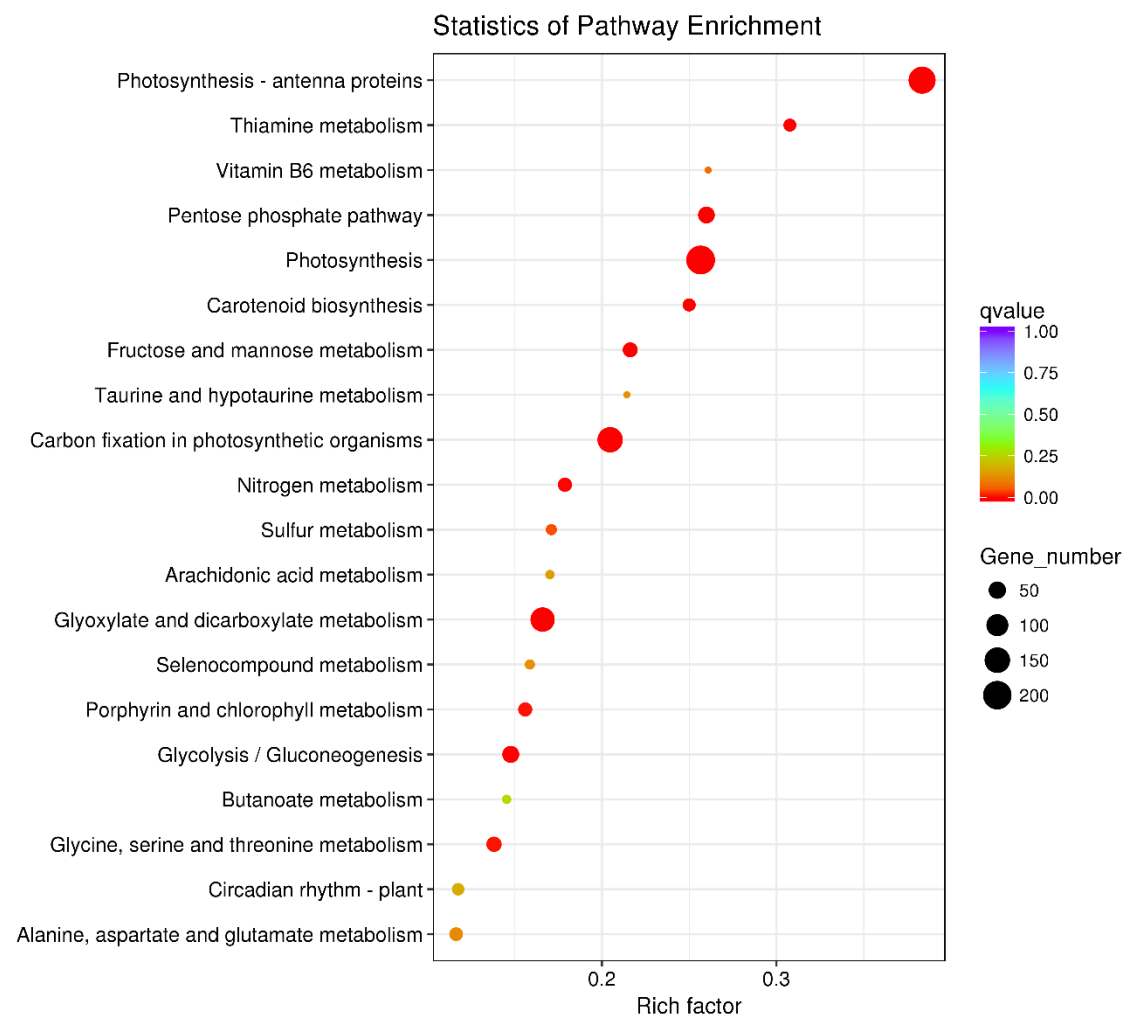

**Additional file 9: KEGG analysis of DEGs down-regulated by HT\_72h and Sen.**

Supplement: Supplementary file 9 — Additional file 9. KEGG analysis of DEGs down-regulated by HT_72h and Sen. [file 12870_2020_2572_MOESM9_ESM.pdf]
