# Supplementary material for: SMRT and Illumina RNA sequencing reveal novel insights into the heat stress response and crosstalk with leaf senescence in tall fescue
Source: BMC Plant Biol. 2020 Aug 3;20:366. doi: 10.1186/s12870-020-02572-4 (PMC7397585; doi:10.1186/s12870-020-02572-4)
Supplement: Supplementary file 10 — Additional file 10. KEGG analysis of DEGs specifically regulated by natural senescence. [file 12870_2020_2572_MOESM10_ESM.pdf]

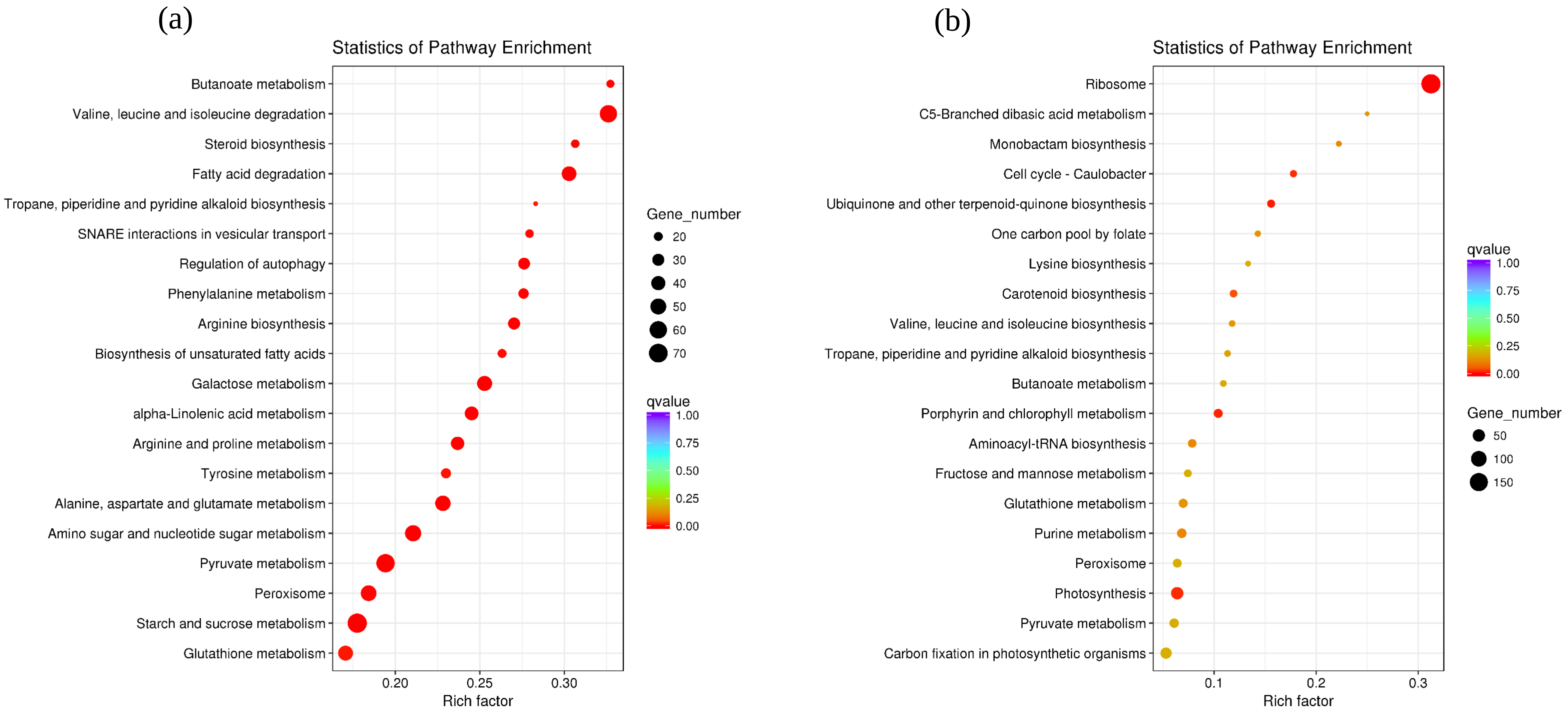

Additional file 10: KEGG analysis of DEGs specifically regulated by natural senescence. (a) The top 20 KEGG pathways of genes specifically up-regulated by Sen. (b) The top 20 KEGG pathways of genes specifically down-regulated by Sen.
