# Supplementary material for: SMRT and Illumina RNA sequencing reveal novel insights into the heat stress response and crosstalk with leaf senescence in tall fescue
Source: BMC Plant Biol. 2020 Aug 3;20:366. doi: 10.1186/s12870-020-02572-4 (PMC7397585; doi:10.1186/s12870-020-02572-4)
Supplement: Supplementary file 13 — Additional file 13. The phenotypic differences between ‘Houndog 5’ (heat-tolerant) and ‘PI535582’ (heat-sensitive) after heat treatments. [file 12870_2020_2572_MOESM13_ESM.pdf]

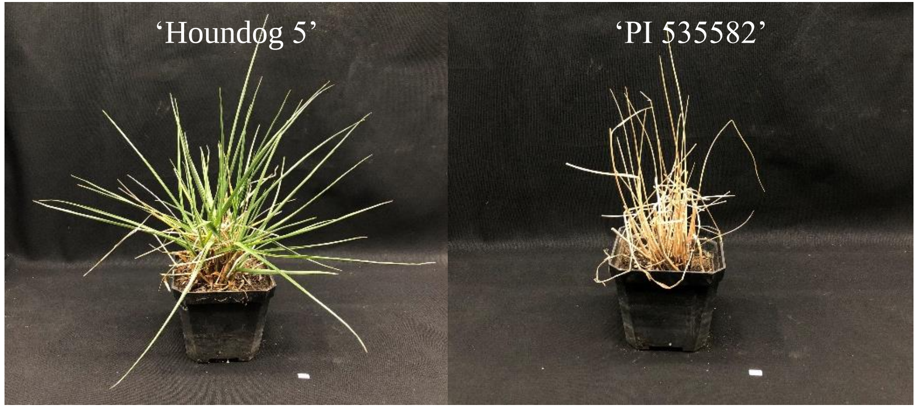

Additional file 13: The phenotypic differences between ‘Houndog 5’ (heat-tolerant) and ‘PI535582’ (heat-sensitive) after heat treatments. The tall fescue plants with the same development state were transferred outside in July 2019 at Wuhan Botanical Garden, CAS. All plants were watered as needed and kept outside until the end of September. The scale = 1cm.
