# Supplementary material for: SMRT and Illumina RNA sequencing reveal novel insights into the heat stress response and crosstalk with leaf senescence in tall fescue
Source: BMC Plant Biol. 2020 Aug 3;20:366. doi: 10.1186/s12870-020-02572-4 (PMC7397585; doi:10.1186/s12870-020-02572-4)
Supplement: Supplementary file 2 — Additional file 2. Functional annotation of the full-length transcriptome in tall fescue. [file 12870_2020_2572_MOESM2_ESM.pdf]

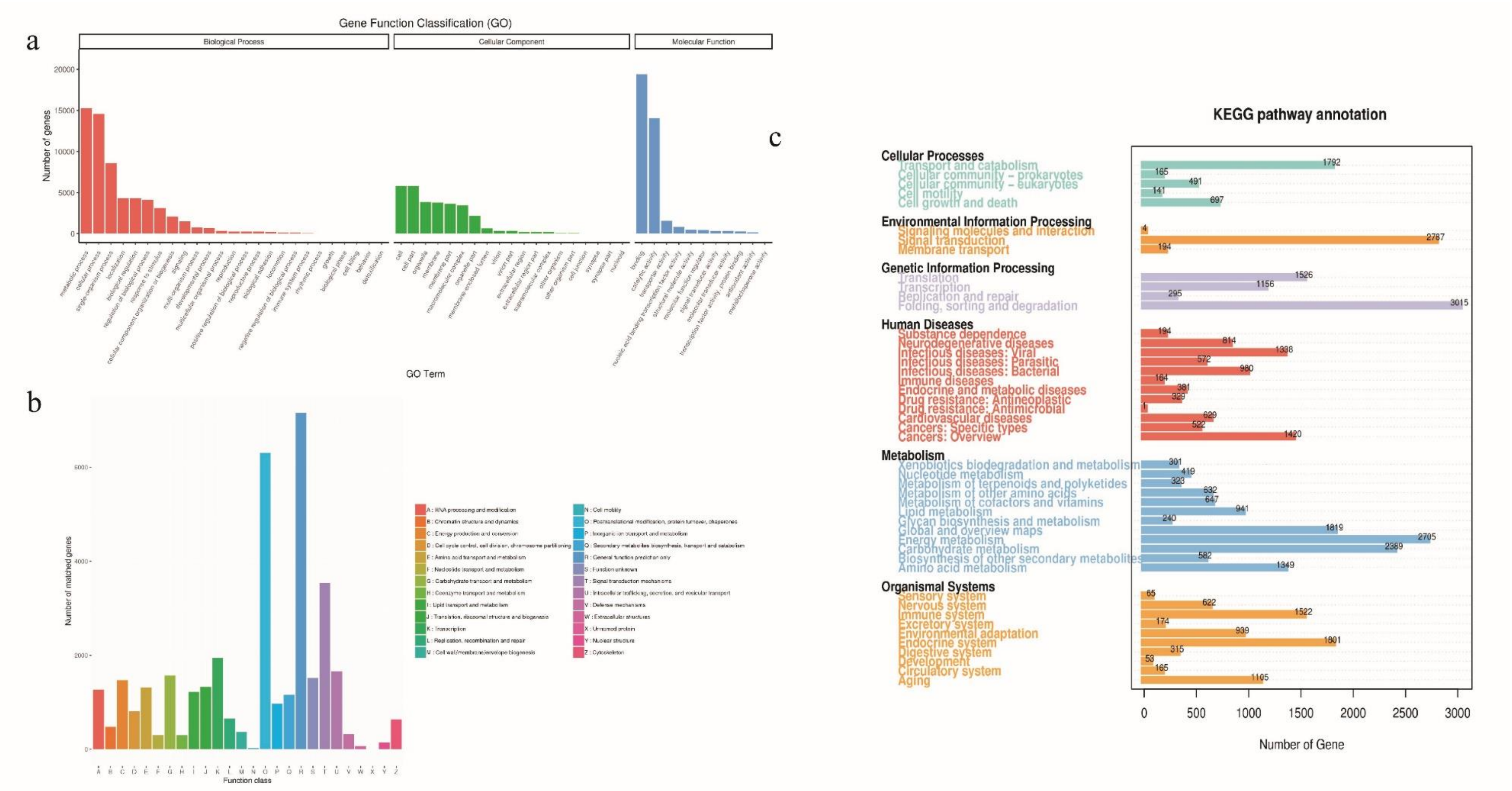

Additional file 2: Functional annotation of full-length transcriptome in tall fescue. (a) Gene ontology (GO) analysis of 31,220 unigenes. (b) Clusters of orthologous groups of proteins (KOG/COG) analysis of 32,982 unigenes. (c) Kyoto encyclopedia of genes and genomes (KEGG) pathway analysis of 50,614 unigenes.
